# M-Sec induced by HTLV-1 mediates an efficient viral transmission

**DOI:** 10.1371/journal.ppat.1010126

**Published:** 2021-11-29

**Authors:** Masateru Hiyoshi, Naofumi Takahashi, Youssef M. Eltalkhawy, Osamu Noyori, Sameh Lotfi, Jutatip Panaampon, Seiji Okada, Yuetsu Tanaka, Takaharu Ueno, Jun-ichi Fujisawa, Yuko Sato, Tadaki Suzuki, Hideki Hasegawa, Masahito Tokunaga, Yorifumi Satou, Jun-ichirou Yasunaga, Masao Matsuoka, Atae Utsunomiya, Shinya Suzu

**Affiliations:** 1 Department of Safety Research on Blood and Biological Products, National Institute of Infectious Diseases, Tokyo, Japan; 2 Joint Research Center for Human Retrovirus Infection, Kumamoto University, Kumamoto, Japan; 3 School of Medicine, University of the Ryukyus, Okinawa, Japan; 4 Department of Microbiology, Kansai Medical University, Osaka, Japan; 5 Department of Pathology, National Institute of Infectious Diseases, Tokyo, Japan; 6 Department of Hematology, Imamura General Hospital, Kagoshima, Japan; 7 Department of Hematology, Rheumatology and Infectious Diseases, Kumamoto University School of Medicine, Kumamoto, Japan; 8 Graduate School of Medical and Dental Sciences, Kagoshima University, Kagoshima, Japan; University of North Carolina at Chapel Hill, UNITED STATES

## Abstract

Human T-cell leukemia virus type 1 (HTLV-1) infects target cells primarily through cell-to-cell routes. Here, we provide evidence that cellular protein M-Sec plays a critical role in this process. When purified and briefly cultured, CD4^+^ T cells of HTLV-1 carriers, but not of HTLV-1^-^ individuals, expressed M-Sec. The viral protein Tax was revealed to mediate M-Sec induction. Knockdown or pharmacological inhibition of M-Sec reduced viral infection in multiple co-culture conditions. Furthermore, M-Sec knockdown reduced the number of proviral copies in the tissues of a mouse model of HTLV-1 infection. Phenotypically, M-Sec knockdown or inhibition reduced not only plasma membrane protrusions and migratory activity of cells, but also large clusters of Gag, a viral structural protein required for the formation of viral particles. Taken together, these results suggest that M-Sec induced by Tax mediates an efficient cell-to-cell viral infection, which is likely due to enhanced membrane protrusions, cell migration, and the clustering of Gag.

## Author summary

In the present study, we identified the cellular protein M-Sec as a host factor necessary for *de novo* infection of human T-cell leukemia virus type 1 (HTLV-1), the causative retrovirus of an aggressive blood cancer known as adult T-cell leukemia/lymphoma. The inhibition or knockdown of M-Sec in infected cells resulted in a reduced viral infection in several culture models and a mouse model. We recently demonstrated a similar role of M-Sec in macrophages infected with another human retrovirus HIV-1, but it has been generally thought that M-Sec is not related to HTLV-1 infection because of the lack of its expression in CD4^+^ T cells, the major target of HTLV-1. In this study, we revealed that CD4^+^ T cells of HTLV-1 asymptomatic carriers, but not those of HTLV-1^-^ individuals, expressed M-Sec, and that the viral protein Tax mediated the induction of M-Sec. Thus, M-Sec is a new and useful tool for further understanding the process of HTLV-1 transmission.

## Introduction

M-Sec (also known as TNF-α-induced protein 2, TNFAIP2) is a key regulator of the formation of plasma membrane protrusions including tunneling nanotubes, the F-actin-containing long plasma membrane extensions [1,2], and plays a critical role in initiating the protrusions or extensions [3,4]. Recent studies have also shown that M-Sec enhances migration, invasion, and metastasis of several cancer cells, such as nasopharyngeal carcinoma [5], breast cancer [6], and esophageal squamous cell carcinoma [7]. M-Sec is a cytosolic protein that shares homology with Sec6, a component of the exocyst complex involved in vesicle trafficking [3]. However, the molecular mechanisms by which M-Sec, which has no known enzymatic activity, regulate membrane protrusions or extensions, and cell motility are largely unknown.

We recently demonstrated that M-Sec promotes cell-to-cell infection of HIV-1 [8,9]. Small molecule compound that inhibits M-Sec-induced membrane protrusions reduced HIV-1 production in monocyte-derived macrophages [8]. Moreover, the knockdown of M-Sec retarded HIV-1 production in U87 glioma cells [9], a widely-used HIV-1 target cell line. As M-Sec inhibition or knockdown reduces membrane protrusions and cell migration in these cells [8,9], M-Sec appears to contribute to the initial phase of HIV-1 transmission by enhancing membrane protrusions and cell migration. However, the role of M-Sec is likely limited to macrophages among the major targets of HIV-1, because M-Sec is expressed in cells of monocytic lineage, but not in CD4^+^ T cells [3,8]. In fact, the widely used CD4^+^ T cell line Jurkat was negative for M-Sec expression, which was the case even after productive HIV-1 replication in the cells [8]. Similarly, M-Sec is thought not to be related to the cell-to-cell transmission of human T-cell leukemia virus type 1 (HTLV-1), another human retrovirus that preferentially infects CD4^+^ T cells.

HTLV-1 infects at least 5–10 million people worldwide [10]. The infection is asymptomatic in most cases, but HTLV-1 causes two distinct diseases: an aggressive blood cancer known as adult T-cell leukemia/lymphoma (ATL), and a neurodegenerative condition known as HTLV-1-associated myelopathy/tropical spastic paraparesis (HAM/TSP). A recent study also demonstrated an increased risk of premature death among HTLV-1-infected individuals, which is independent of ATL and HAM/TSP [11]. Cell-to-cell infection is recognized as a central route for the transmission of HTLV-1 because the cell-free infection is inefficient [12–16]. Thus, it is important to fully understand the process of cell-to-cell infection.

Here, we report that, in contrast to HIV-1, HTLV-1 induces M-Sec expression in CD4^+^ T cells, and M-Sec contributes to efficient cell-to-cell infection of HTLV-1.

## Results

### HTLV-1 induces M-Sec in CD4^+^ T cells

**Fig 1:**
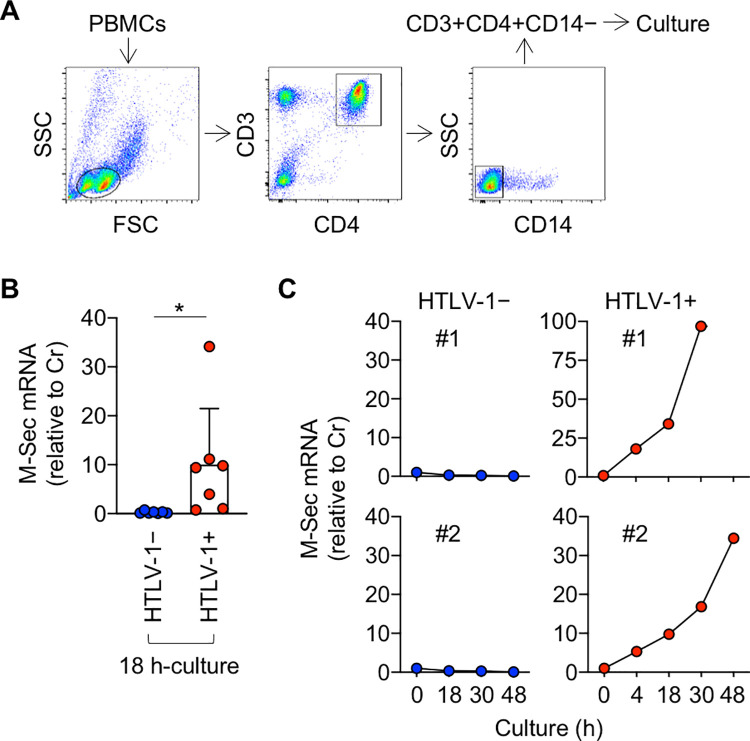
Expression of M-Sec in cultured CD4^+^ T cells of HTLV-1 carriers. (**A**) The CD3^+^CD4^+^CD14^-^ cells in the live cell gate were sorted from peripheral blood mononuclear cells (PBMCs). The profile of an HTLV-1 carrier is shown as an example. CD14 was used to exclude monocytes, which abundantly express M-Sec [3,8]. (**B**) The sorted CD3^+^CD4^+^CD14^-^ cells of HTLV-1^-^ individuals (HTLV-1−; n = 7) or HTLV-1 carriers (HTLV-1+; n = 7) were analyzed for the expression of M-Sec mRNA by using qRT-PCR, before or after culturing for 18 h. The expression levels are shown by setting the value of un-cultured cells as 1. *p < 0.05. (**C**) The sorted CD3^+^CD4^+^CD14^-^ cells of HTLV-1^-^ individuals (HTLV-1−; #1 and #2) or HTLV-1 carriers (HTLV-1+; #1 and #2) were cultured for the indicated periods and analyzed as in (**B**). The expression levels are shown by setting the value of un-cultured cells as 1.

Recent studies have revealed that the transcription of HTLV-1 provirus is regulated by a unique mechanism⁠. Viral plus-strand transcription is silent in freshly isolated cells of HTLV-1 carriers, but a short-term *ex vivo* culture induces a spontaneous transcriptional burst of viral genes, including the gene encoding the trans-activating protein Tax [17,18]. In this study, we found that when sorted and cultured (Fig 1A), CD4^+^ T cells of most HTLV-1 carriers tested expressed M-Sec (Fig 1B). Such a change was not observed in CD4^+^ T cells of HTLV-1^-^ individuals. M-Sec induction in CD4^+^ T cells of carriers was detected as early as 4 h after the beginning of culture and increased at least up to 48 h (Fig 1C). Although we analyzed carriers whose viral load was similar (6.3–13.1%, 9.3 ± 2.6%, n = 7), M-Sec induction was weak or undetected in several carriers (Fig 1B). Similar variability was observed in viral transcripts among HTLV-1^+^ individuals [19].

**Fig 2:**
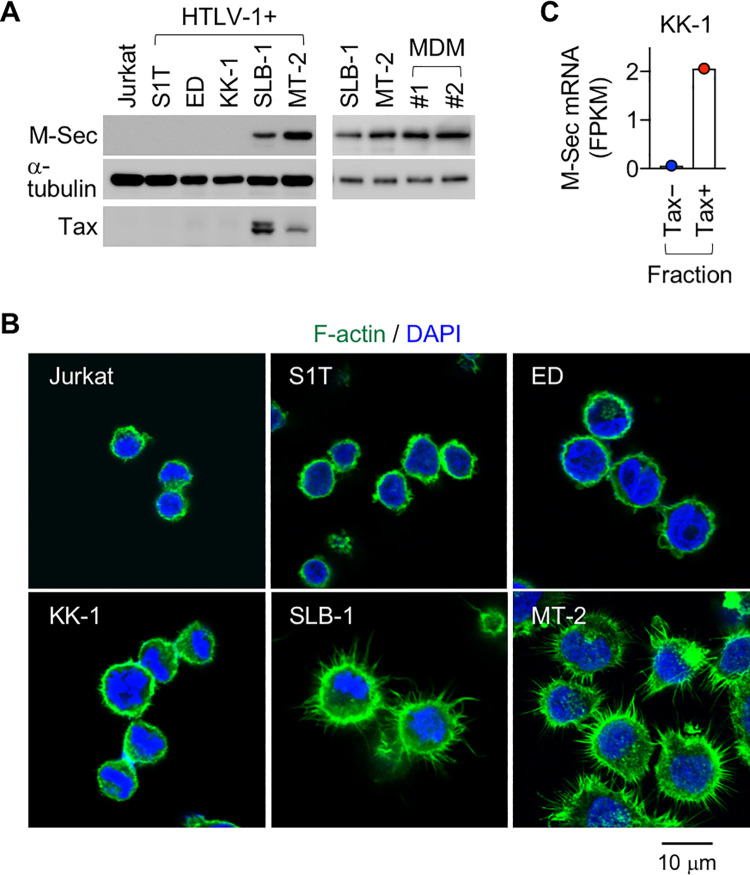
Expression of M-Sec in Tax^+^ T cells. (**A**) The indicated T cell lines were analyzed for the expression of M-Sec- and Tax protein by western blotting. α-tubulin blot is the loading control. Monocyte-derived macrophages (MDM) obtained by culturing monocytes from healthy volunteers (#1 and #2) were added as a positive control for M-Sec. (**B**) The indicated T cell lines were co-stained with phalloidin (green, to visualize F-actin) and DAPI (blue). Scale bar: 10 μm. (**C**) KK-1 cells expressing GFP in a Tax-dependent manner were subjected to sorting (Tax^-^ or Tax^+^ fraction) followed by RNA-seq (accession number in NCBI GEO database: GSE108601) [18]. The RNA-seq data were analyzed for the expression of M-Sec mRNA in those fractions. FPKM, Fragments Per Kilobase of exon per Million mapped reads.

Furthermore, HTLV-1^+^Tax^+^ T cell lines (SLB-1 and MT-2) expressed M-Sec protein, the level of which was comparable to that of monocyte-derived macrophages (Fig 2A), which are typical M-Sec^+^ cells [3,8]. SLB-1 and MT-2 cells also formed many membrane protrusions (Fig 2B), the hallmark activity of M-Sec [3]. The HTLV-1^-^ cell line (Jurkat) and HTLV-1^+^Tax^-^ cell lines (S1T, ED, and KK-1) did not show clear M-Sec expression and membrane protrusions. Interestingly, KK-1 cells expressed Tax, but the number of Tax^+^ KK-1 cells in the culture was small at any given time because of the sporadic on/off switching of Tax [18] (S1 Fig). The minor Tax^+^ fraction, but not the major Tax^-^ fraction, highly expressed M-Sec (Fig 2C). Moreover, in a mouse model of HTLV-1 infection [20], in which humanized mice were inoculated with MT-2 cells, only Tax^high^ human CD3^+^ cells expressed M-Sec at a detectable level (S1 Table, mouse #3 and #4).

**Fig 3:**
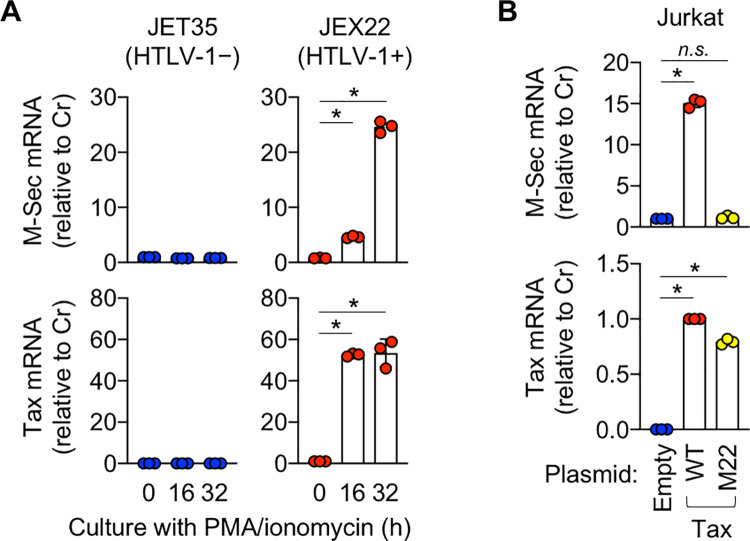
Induction of M-Sec expression by Tax in Jurkat cells. (**A**) The control JET35 (left) or HTLV-1-infected JEX22 cells (right) were left un-stimulated or stimulated with PMA and ionomycin for 16 or 32 h, and analyzed for the expression of M-Sec- (upper) and Tax mRNA (lower) by qRT-PCR. The expression levels are shown by setting the value of un-stimulated cells as 1 (n = 3). *p < 0.05. (**B**) Jurkat cells were nucleofected with the empty vector, or Tax plasmid expressing wild type (WT) or M22 mutant. After 24 h, the cells were analyzed for the expression of M-Sec- (upper) and Tax mRNA (lower) by qRT-PCR. M-Sec expression levels are shown by setting the value of cells nucleofected with the empty vector as 1 (n = 3). Tax expression levels are shown by setting the value of cells nucleofected with the wild type Tax as 1 (n = 3). *p < 0.05. *n*.*s*., not significant.

To further analyze M-Sec expression by HTLV-1, we used JEX22, a Jurkat cell-based HTLV-1-infected line that produces HTLV-1 upon stimulation with phorbol 12-myristate 13-acetate (PMA) and ionomycin [20]. When stimulated, JEX22 cells expressed Tax and M-Sec (Fig 3A, right). Such a change was not observed in uninfected control cells (JET35; Fig 3A, left). When expressed in Jurkat cells, Tax induced M-Sec (Fig 3B). Interestingly, a Tax mutant M22, which can activate the HTLV-1 LTR promoter but not the NF-κB promoter [21] (S2 Fig), failed to induce M-Sec (Fig 3B). Collectively, these results suggest that Tax, which is expressed by the viral plus-strand transcriptional burst, induces M-Sec in CD4^+^ T cells through potent activation of the NF-κB pathway.

### M-Sec mediates efficient HTLV-1 infection in co-cultures

**Fig 4:**
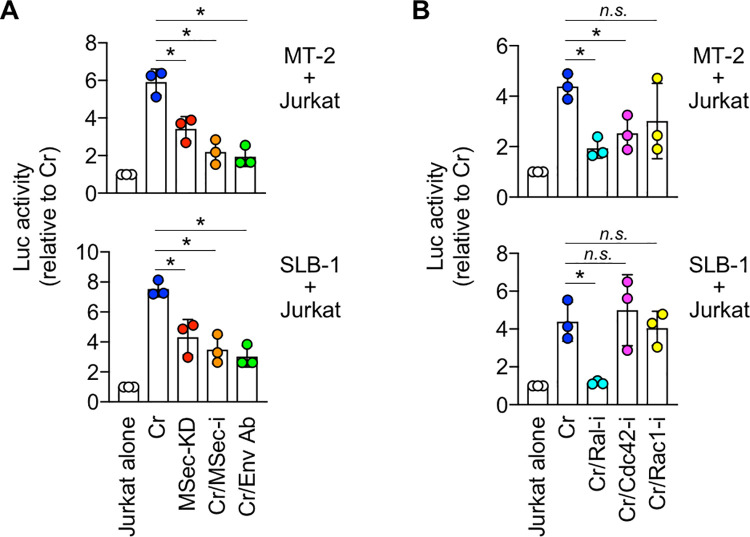
Effect of M-Sec knockdown/inhibition on viral infection in co-culture using cell lines. (**A**) Reporter Jurkat cells were cultured alone (Jurkat alone), or co-cultured with the control (Cr) or M-Sec knockdown (MSec-KD) MT-2- or SLB-1 cells for 16 h. In assays in which the effect of M-Sec inhibitor (MSec-i) was tested, control MT-2- or SLB-1 cells were pre-treated with MSec-i for 48 h and used for the co-culture (Cr/MSec-i). The anti-gp46 Env neutralizing antibody was included as a reference (Cr/Env Ab). The luciferase activities are shown by setting the value of Jurkat alone as 1 (n = 3). *p < 0.05. (**B**) The control MT-2- or SLB-1 cells were pre-cultured in the absence (Cr) or presence of Ral inhibitor (Cr/Ral-i), Cdc42 inhibitor (Cr/Cdc42-i), or Rac1 inhibitor (Cr/Rac1-i) for 48 h, and used for the co-culture with Jurkat cells. The luciferase activities are shown by setting the value of Jurkat alone as 1 (n = 3). *p < 0.05.

We performed co-culture assays to examine how M-Sec contributes to HTLV-1 infection. MT-2 or SLB-1 was used as infected cells, and Jurkat carrying the HTLV-1 LTR promoter-driven luciferase gene was used as the target cells. M-Sec knockdown MT-2 or SLB-1 bulk culture cells were prepared (MSec-KD; S3 Fig), and M-Sec inhibitor was also used (MSec-i; S3 Fig). It has been shown that MSec-i reduces the formation of tunneling nanotubes in several cell lines engineered to express M-Sec [8,22] and the production of HIV-1 in the culture of macrophages without affecting podosome formation and phagocytic activity of the cells [8,23]. Control MT-2 or SLB-1 cells were prepared by transducing non-targeting shRNA (Cr; S3 Fig). We found that M-Sec knockdown in MT-2 or SLB-1 cells, or MSec-i addition to control MT-2 or SLB-1 cells reduced viral infection to target cells (Fig 4A), albeit modestly when compared to the anti-envelope (Env) antibody [24] (Cr/Env Ab).

A group of small GTPases is a possible downstream effector of M-Sec, as both small GTPases and M-Sec regulate actin cytoskeleton remodeling [3,6,25]. Among the inhibitors of small GTPases tested [26,27], a Cdc42 inhibitor (ZCL278) reduced viral infection to target cells in MT-2-based co-culture (Fig 4B, upper), whereas a Ral inhibitor (BQU57) reduced viral infection in both MT-2- and SLB-1-based co-cultures (Fig 4B). This result was consistent with the finding that M-Sec-induced membrane protrusions in macrophage-like RAW264 cells were strongly inhibited by a dominant-negative Ral, and modestly inhibited by a dominant-negative Cdc42 [3].

**Fig 5:**
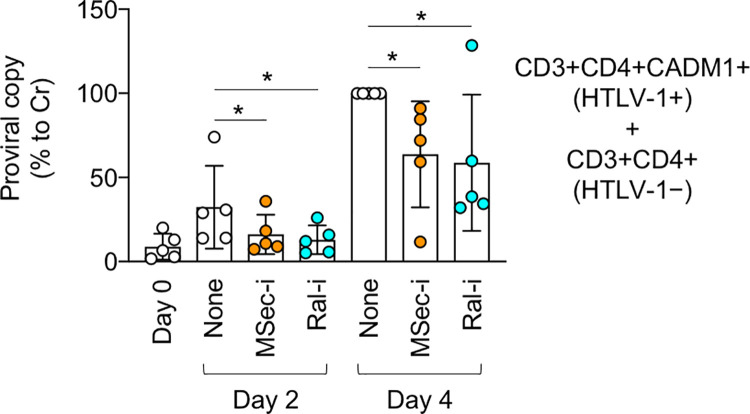
Effect of M-Sec inhibition on viral infection in co-culture using primary cells. The CD3^+^CD4^+^CADM1^+^ cells in the live cell gate were sorted from the PBMCs of HTLV-1 carriers (n = 5). The CD3^+^CD4^+^ cells were also sorted from PBMCs of HTLV-1^−^ individuals as target cells. They were mixed and co-cultured in the absence (None) or presence of M-Sec inhibitor (MSec-i) or Ral inhibitor (Ral-i) for 2 or 4 days. The number of proviral copies in the co-culture was quantified using qPCR and is shown as percentages relative to that of the co-culture with no inhibitor on day 4 (the third bar from the right). *p < 0.05.

The results of co-cultures using cell lines prompted us to test the effect of the M-Sec inhibitor (MSec-i) and Ral inhibitor (Ral-i) on a primary cell-based co-culture. HTLV-1-infected CD4^+^ T cells were enriched in the CADM1^+^ fraction [28]. When sorted and cultured (S4A Fig), approximately half of the CD3^+^CD4^+^CADM1^+^ cells of carriers expressed M-Sec at a detectable level, albeit modestly when compared to monocytes (S4B and S4C Fig). Thus, we co-cultured the CD3^+^CD4^+^CADM1^+^ cells of carriers and the CD3^+^CD4^+^ cells of HTLV-1^-^ individuals as target cells, and confirmed that both M-Sec inhibitor and Ral inhibitor reduced the number of proviral copies in the co-culture (Fig 5). The timing (day 2 or 4) and extent of the inhibitory effect of those inhibitors were variable among carriers (S5 Fig), as observed with the M-Sec induction in CD4^+^ T cells of carriers (Fig 1B). As an obvious increase in the number of sorted CD3^+^CD4^+^CADM1^+^ cells during the culture period was not observed in a microscopic analysis, the increase in the number of proviral copies might mainly reflect *de novo* infection. M-Sec knockdown did not affect the proliferation of MT-2 or SLB-1 cells, and M-Sec inhibitor or Ral inhibitor did not show any cytotoxicity to MT-2, SLB-1, Jurkat or primary CD4^+^ T cells at the concentration used (S6 Fig). Thus, the results of different co-culture systems suggest that M-Sec mediates efficient cell-to-cell HTLV-1 infection.

### M-Sec mediates efficient HTLV-1 infection in a mouse model

**Fig 6:**
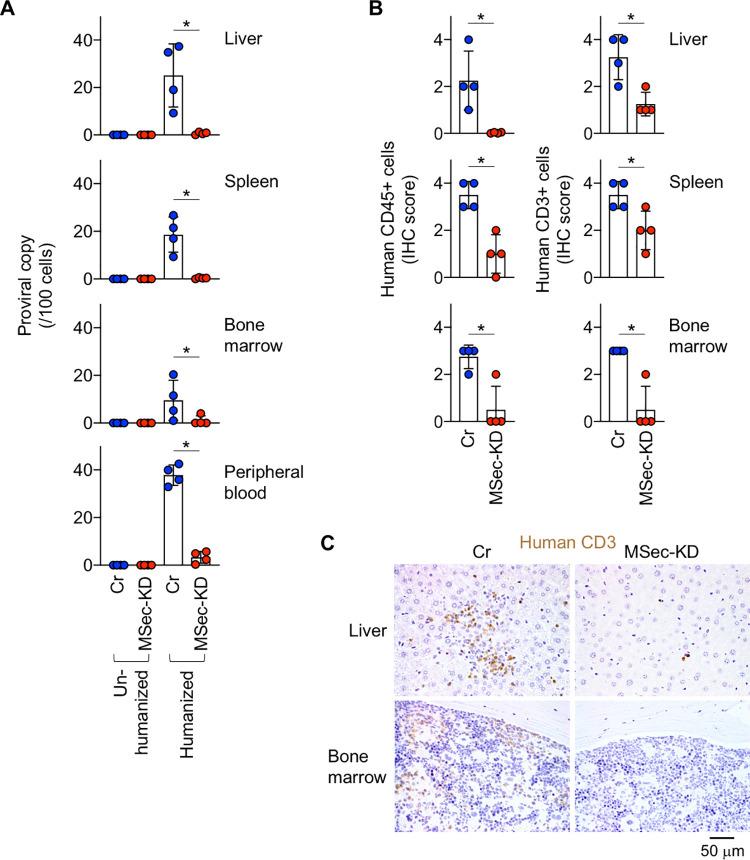
Effect of M-Sec knockdown on viral infection in a mouse model. (**A**) The un-humanized or humanized mice were inoculated intraperitoneally with irradiated control (Cr)- or M-Sec knockdown (MSec-KD) MT-2 cells. After 4 weeks, the cells in the liver, spleen, bone marrow, or peripheral blood were analyzed for proviral copies by qPCR (n = 4 for each group). The numbers of proviral copies per 100 cells are shown. *p < 0.05. (**B**, **C**) The humanized mice were inoculated with irradiated MT-2 cells as in (**A**). After 4 weeks, the liver, spleen, or bone marrow was analyzed for human CD45^+^ or human CD3^+^ cells by immunohistochemistry (IHC). In (**B**), semi-quantitative scores are shown (n = 4 for each group). *p < 0.05. In (**C**), typical images of human CD3^+^ cells (brown) in the liver or bone marrow are shown. Scale bar: 50 μm.

To examine how M-Sec contributes to *in vivo* HTLV-1 infection, we next utilized a mouse model [20]. When un-humanized immunodeficient mice were inoculated intraperitoneally with control- or M-Sec knockdown (MSec-KD) MT-2 cells, the number of MT-2 cells in the spleen did not differ between the two groups (S7 Fig). Under these conditions, the provirus in the tissues, including the spleen, was below the detection limit (Un-humanized; Fig 6A). However, once humanized immunodeficient mice were used, the provirus became detectable (Humanized; Fig 6A), indicating *de novo* infection of reconstituted human T cells. Of note, under these conditions, the number of proviral copies in the liver, spleen, bone marrow, and peripheral blood of the M-Sec knockdown group were lower than that of the control group (Fig 6A). In mouse models of HTLV-1 infection, the number of human cells in tissues correlated with that of proviral copies [29,30]. For instance, Percher et al. reported the proliferation of infected human T cells in spleens of MT-2-inoculated humanized mice, the extent of which correlated with the level of plasma viral load [30]. Consistent with this finding, the number of human CD45^+^ cells or CD3^+^ T cells in the liver, spleen, and bone marrow of the M-Sec knockdown group were lower than that of the control group (Fig 6B and 6C). These results further support the idea that M-Sec mediates efficient cell-to-cell HTLV-1 infection.

### M-Sec not only enhances membrane protrusions and cell migration, but also regulates the intracellular distribution of Gag

**Fig 7:**
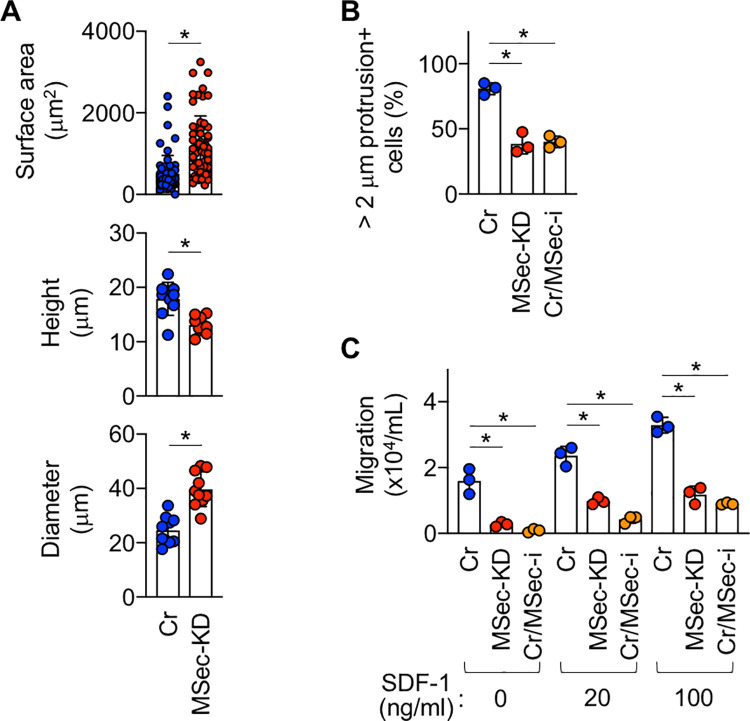
Effect of M-Sec knockdown/inhibition on morphology, membrane protrusions, and migration of MT-2 cells. (**A**) The control (Cr)- or M-Sec knockdown (MSec-KD) MT-2 cells were stained with phalloidin (to visualize F-actin) and analyzed for the surface area (top, 60 cells for each), height (middle, 10 cells for each), or diameter (bottom, 10 cells for each). *p < 0.05. (**B**) The control (Cr)- or M-Sec knockdown (MSec-KD) MT-2 cells were stained with phalloidin (to visualize F-actin) analyzed for membrane protrusions. Control cells pre-treated with M-Sec inhibitor for 48 h were also added (Cr/MSec-i). Three different fields were randomly selected, and the percentages of membrane protrusions^+^ cells were quantified. *p < 0.05. (**C**) The control (Cr)- or M-Sec knockdown (MSec-KD) MT-2 cells were analyzed for migratory activity by using transwell assay. Control cells pre-treated with M-Sec inhibitor (MSec-i) for 48 h were also added. The migration toward SDF-1 (20 or 100 ng/mL) was also assessed. The numbers of cells that migrated through the inserts into lower wells were enumerated by the trypan blue dye exclusion method (n = 3). *p < 0.05.

We next examined how M-Sec contributes to *in vitro* and *in vivo* HTLV-1 infection. M-Sec has been reported to enhance the proliferation of several cancer cells [6,7]. However, we did not find any inhibitory effect of M-Sec knockdown on the proliferation of MT-2 cells (S6A and S8 Figs), which was the case for SLB-1 cells (S6A Fig) and macrophage-like RAW264 cells (S9 Fig). Instead, M-Sec knockdown in MT-2 cells caused morphological changes, as evidenced by an increase in the surface area and shorter height/longer diameter (Fig 7A). M-Sec knockdown or inhibition also reduced plasma membrane protrusions (Fig 7B) and the migratory activity of MT-2 cells (Fig 7C). If a cell had one or more F-actin^+^ protrusions longer than 2 μm, it was considered protrusion^+^ (Fig 7B). The migration of MT-2 cells toward the chemokine SDF-1/CXCL12 was also impaired by M-Sec knockdown or inhibition (Fig 7C).

**Fig 8:**
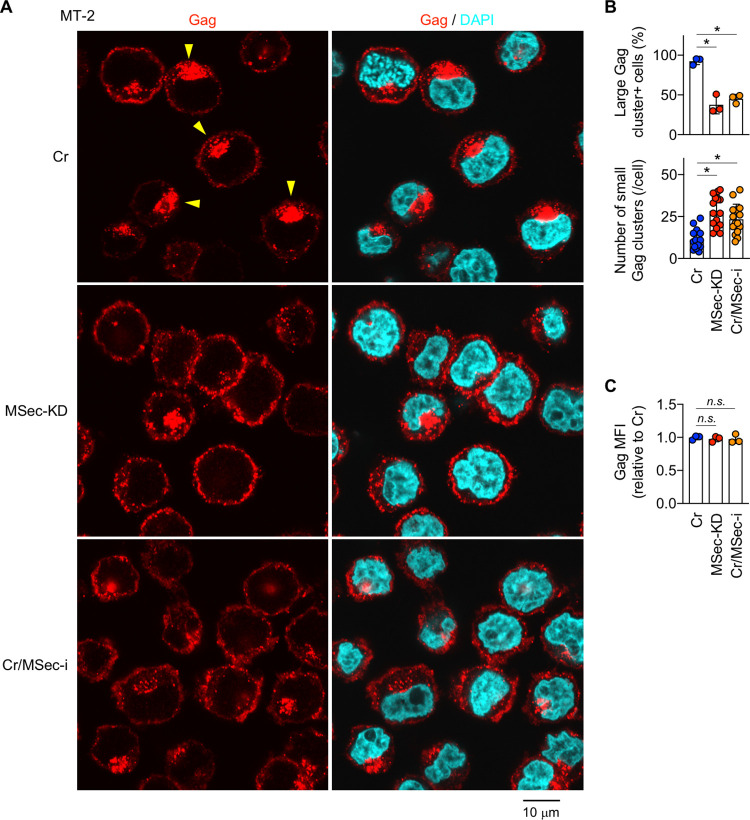
Effect of M-Sec knockdown/inhibition on the intracellular distribution of Gag in MT-2 cells. (**A**) The control (Cr)- or M-Sec knockdown (MSec-KD) MT-2 cells, or control cells pre-treated with M-Sec inhibitor for 48 h (Cr/MSec-i) were analyzed for Gag (red). The nuclei were also stained with DAPI (blue). Yellow arrowheads (top left panel) indicate the large Gag clusters. Scale bar: 10 μm. (**B**) Cells were analyzed as in (**A**). Three different fields were randomly selected, and the percentages of the large Gag cluster^+^ cells were quantified (upper). The numbers of small clusters of Gag per cell are also shown (lower, 16 cells for each). *p < 0.05. (**C**) Cells prepared as in (**A**) were analyzed for Gag expression by flow cytometry. The mean fluorescence intensities (MFI) of Gag are shown by setting the value of M-Sec inhibitor-free control cells as 1 (n = 3). *n*.*s*., not significant.

**Fig 9:**
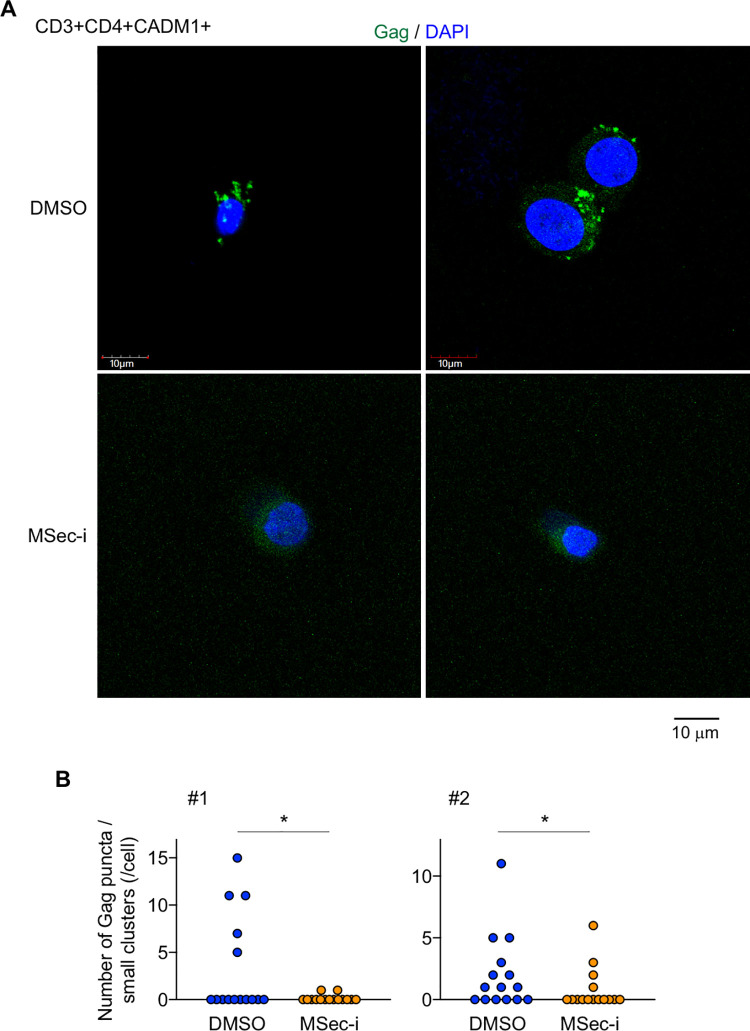
Effect of M-Sec inhibition on the intracellular distribution of Gag in CD3^+^CD4^+^CADM1^+^ T cells of HTLV-1 carriers. (**A**) The CD3^+^CD4^+^CADM1^+^ cells in the live cell gate were sorted from PBMCs of an HTLV-1 carrier, cultured with vehicle (DMSO, upper panels) or M-Sec inhibitor (MSec-i, lower panels) for 3 days, and analyzed for Gag (green). The nuclei were also stained with DAPI (blue). Scale bar: 10 μm. (**B**) The sorted CD3^+^CD4^+^CADM1^+^ cells of HTLV-1 carriers (#1 and #2) were analyzed as in (**A**). The numbers of puncta or small clusters of Gag per cell are shown (16 cells for each). *p < 0.05.

The enhanced migration and membrane protrusions of infected cells may increase the likelihood of encountering target cells and contact between those cells, thereby contributing to efficient cell-to-cell infection. However, these well-known functions of M-Sec might not be sufficient to explain the potent effect of M-Sec knockdown in the mouse model (Fig 6). Gag is a viral structural protein that localizes to the inner leaflet of the plasma membrane [12,31–35]. In both MT-2 and SLB-1 cells, the signal of Gag did not overlap with that of Calnexin, an endoplasmic reticulum marker (S10 Fig). The signal of Gag partially overlapped with that of GM130 (a Golgi marker) in MT-2 cells, but not in SLB-1 cells (S10 Fig). Thus, Gag appears to mainly localize to the inner leaflet of the plasma membrane in these cells. Gag is also known to form puncta, which is an indicator of their assembly requisite for the formation of viral particles [12,31–35]. In fact, both control (S1 Video) and M-Sec knockdown MT-2 cells (S2 Video) had many puncta. Of note, we found that control MT-2 cells had a large cluster of Gag in addition to those puncta (Fig 8A, yellow arrowheads for large clusters), and that M-Sec knockdown (MSec-KD) or inhibition (Cr/MSec-i) reduced the percentage of large Gag cluster^+^ cells (Fig 8A and 8B, upper). This change was associated with an increase in the number of small clusters of Gag per cell (Fig 8B, lower), which was consistent with an unchanged total amount of Gag per cell (Fig 8C). It was likely that puncta grew into a large cluster by recruiting additional puncta because the large cluster was composed of many puncta (S11 Fig). M-Sec knockdown SLB-1 cells also showed a reduced percentage of large Gag cluster^+^ cells (S12A and S12B Fig, upper) and increased number of small clusters of Gag per cell (S12B Fig, lower). These results suggest that M-Sec mediates an efficient clustering of Gag. Consistent with the idea, when CADM1^+^ T cells of HTLV-1 carriers were analyzed, the number of puncta or small clusters of Gag per cell in M-Sec inhibitor-treated cultures was lower than that of the control cultures (Fig 9A and 9B).

## Discussion

It has been believed that M-Sec is not related to an infection in T cells because of the lack of their expression under physiological conditions. In this study, we revealed that M-Sec plays a critical role in HTLV-1 infection in CD4^+^ T cells. Our study suggests that Tax expressed by the viral plus-strand transcriptional burst induces M-Sec through a potent activation of NF-κB pathway, and that M-Sec mediates an efficient cell-to-cell infection of HTLV-1 likely due to enhanced membrane protrusions, cell migration, and the clustering of Gag.

We demonstrated that M-Sec is a Tax-inducible protein. A series of experiments using CD4^+^ T cells from HTLV-1 carriers (Fig 1), HTLV-1^+^ cell lines, including SLB-1, MT-2, and KK-1 (Fig 2), and Tax-expressing Jurkat cells (Fig 3) support the idea that Tax translated by the viral plus-strand transcriptional burst induces the expression of M-Sec in CD4^+^ T cells. Tax-mediated M-Sec induction required activation of the NF-κB pathway (Fig 3B). Tax is known to activate NF-κB [36], while the effects of HIV-1 on NF-κB activation depend on the type of cells or their activation state [37], which explains why M-Sec was not induced in HIV-1-infected Jurkat cells despite active viral replication [8]. The finding that LMP-1, an oncoprotein of Epstein-Barr virus, upregulates M-Sec in nasopharyngeal carcinoma cells through NF-κB activation [5] also supports M-Sec induction in CD4^+^ T cells by the Tax-NF-κB cascade.

Tax is essential for *de novo* infection of HTLV-1 as it induces the expression of viral genes, but it simultaneously allows the recognition of infected cells by cytotoxic T lymphocytes [38]. Thus, the transient or intermittent expression of Tax by the viral plus-strand transcriptional burst [17,18] may be a strategy for HTLV-1 to maintain a balance between escape from the immune system and *de novo* infection [38]. To ensure viral infection within its limited period of expression, Tax may induce cellular proteins including M-Sec, which mediates an efficient transmission of HTLV-1 as demonstrated by our analyses of co-cultures (Figs 4 and 5) and the mouse model (Fig 6).

The ability of M-Sec to enhance migratory activity of infected cells (Fig 7C) can increase the likelihood of encountering target cells, thereby contributing to an efficient cell-to-cell infection. In fact, among Tax-inducible cellular proteins that facilitate contact between HTLV-1-infected cells and target cells [39–41], Gem, a member of the small GTP-binding proteins, not only enhances the migratory activity of infected cells but also mediates an efficient viral infection from infected cells to target cells in a co-culture assay [41]. The ability of M-Sec to enhance the formation of membrane protrusions (Fig 7B) can facilitate contact between the infected cells and target cells. The protrusions of MT-2 or SLB-1 cells (Fig 2B) appear to be smaller in length but larger in number than those of HIV-1-infected macrophages or U87 cells [8,9]. Furthermore, the ability of M-Sec to facilitate the clustering of Gag (Figs 8, S12, and 9) may be beneficial for viral transmission because Gag is the key driver for the formation of viral particles [31–35]. Consistent with this idea, an alanine-scanning mutagenesis analysis of Gag has identified several mutants that not only fail to form puncta but also produce lesser amounts of viral particles when compared to the wild type Gag [34].

M-Sec inhibition in macrophages or knockdown in U87 glioma cells reduced the production of HIV-1 [8,9]. However, such viral reduction became less obvious over time in the culture [9], implying that M-Sec mainly contributes to the initial phase of HIV-1 transmission. In the co-culture of the present study, reduced viral transfer from M-Sec knockdown MT-2 cells to Jurkat cells was observed at 16 h (Fig 4A, upper), but not at 36 h (S13 Fig). In addition, in several cases in the co-culture using CADM1^+^ T cells of HTLV-1 carriers, the reduced proviral copies by M-Sec inhibition found at 2 days were lost at 4 days (S5 Fig, #4 and #5). Meanwhile, the extent of the reduced proviral copies by M-Sec knockdown in the mouse model found at 4 weeks (Fig 6) appears to be more obvious than that in the co-cultures. Thus, to clarify whether M-Sec mainly contributes to the initial phase of HTLV-1 transmission, detailed time-course analyses will be necessary for the assays involving the mouse model.

M-Sec expression in several cancer cells enhances their invasion/metastasis [5–7] or proliferation [6,7]. In our cultures, M-Sec knockdown or inhibition in MT-2 cells reduced their migration (Fig 7C), but not proliferation (S6 and S8 Figs). When intraperitoneally injected into immunodeficient mice, the numbers of M-Sec knockdown MT-2 cells in the spleen were not different from those of the control MT-2 cells (S7 Fig). These results suggest that M-Sec knockdown does not affect the proliferation or survival of MT-2 cells in mice or the migration from the peritoneal cavity to the spleen. Thus, it is tempting to speculate that a weak intra-tissue migration of M-Sec knockdown MT-2 cells and their reduced membrane protrusions/Gag clustering could explain the reduced viral infection in the mouse model, although further studies including the identification of M-Sec mutants which lack selected functions are necessary to prove this hypothesis.

M-Sec regulates cellular morphology, migration, and membrane protrusions [3–9]. In addition to these well-known abilities, our current study suggests that M-Sec functions as a regulator of HTLV-1 Gag clustering. M-Sec binds phosphatidylinositol (4,5)-bisphosphate (PI(4,5)P_2_) [4], but this feature may not explain the newly identified function, since the binding of HTLV-1 Gag to cellular membranes is essentially independent of PI(4,5)P_2_ [32,33]. Thus, the strong ability of M-Sec to induce membrane deformation and actin cytoskeleton remodeling [3–6] may explain the regulation of HTLV-1 Gag clustering. Alternatively, cellular protein(s) involved in vesicle trafficking that have been predicted to interact with M-Sec in the STRING database [42,43], or Ral, which is the possible downstream effector of M-Sec [3], may be attributed to the role of M-Sec in HTLV-1 Gag clustering. Unlike HTLV-1 Gag, HIV-1 Gag binds PI(4,5)P_2_ [32,33]. Thus, it will be intriguing to test whether M-Sec affects the clustering of HIV-1 Gag.

How different M-Sec functions are related to each other is still unclear. How these functions contributes to HTLV-1 transmission and the extent to which each function contributes remain unexplored. Despite these unresolved questions, the present study revealed the importance of M-Sec for HTLV-1 transmission. M-Sec is a new and useful tool to further clarify the process of cell-to-cell infection of HTLV-1.

## Materials and methods

### Ethics statement

All protocols involving human subjects were reviewed and approved by the institutional review board of Kumamoto University. The protocols were reviewed and approved by the ethical committee of Imamura General Hospital. Written informed consent was obtained from all subjects in accordance with the Declaration of Helsinki. The research protocols for mouse experiments were approved by the animal center and the ethical committee of the National Institute of Infectious Diseases and were performed according to the institutional guidelines for the experimental use of animals.

### CD4^+^ T cells of HTLV-1 carriers and HTLV-1^-^ individuals

Peripheral blood mononuclear cells (PBMCs) of asymptomatic HTLV-1 carriers (proviral load: 6.3–13.1%) and HTLV-1^-^ individuals were used in this study. CD4^+^ T cells in the live cell gate were sorted from the PBMCs by using a FACSAria II (BD Biosciences) and cultured in RPMI 1640 medium containing 10% FCS and 10 ng/mL rhIL-2 (BioLegend). The antibodies used for staining were as follows: PE-anti-CD3 (OKT3), Pacific Blue-anti-CD3 (OKT3), APC-anti-CD4 (RPA-T4), Pacific Blue-anti-CD14 (M5E2; all from BioLegend), and PE-anti-CADM1 (3E1; MBL, Nagoya, Japan). Total RNA and genomic DNA were isolated using the RNeasy micro kit and QIAamp DNA micro kit (both from Qiagen), respectively. The RNA and DNA samples were used for qRT-PCR (M-Sec mRNA) and qPCR (provirus), respectively. The cells were also used for immunofluorescence (Gag).

### HTLV-1^+^ T cell lines

MT-2 cells were obtained from the Japanese Collection of Research Bioresources Cell Bank. S1T, KK-1, and SLB-1 cells were provided by N. Arima (Kagoshima University, Japan), H. Hasegawa (Nagasaki University, Japan), and M. Fujii (Niigata University, Japan), respectively. All the cells were maintained in RPMI 1640 medium containing 10% FCS. KK-1 cells were cultured in the presence of 10 ng/mL rhIL-2. The RNA-seq data of KK-1 cells expressing GFP in a Tax-dependent manner (accession number in NCBI GEO database: GSE108601) [18] were also analyzed.

### M-Sec knockdown in MT-2- and SLB-1 cells

The knockdown of M-Sec in MT-2 or SLB-1 cells was performed using MISSION shRNA lentiviral transduction particles (Sigma). pLKO.1-puro-CMV-tGFP shRNA targeting human M-Sec (TRCN0000330220) or a scrambled nontargeting control was used. Control cells expressing non-targeting siRNA or cells expressing M-Sec-targeting siRNA were selected under the culture containing 0.3 μg/mL puromycin and used without cloning to exclude the possibility of clonal variation. GFP expression in these cells and reduced M-Sec expression in M-Sec knockdown cells were confirmed using flow cytometry and western blotting, respectively.

### Jurkat cells

The Jurkat cell-based JEX22 cell line and its parental JET35 cell line [20,44] were used. JEX22 had been infected with the HTLV-1 molecular clone pX1 MT-M [45] and produced HTLV-1 upon stimulation with 50 ng/mL PMA and 1 μM ionomycin [20]. Jurkat cells (clone E6-1) were obtained from the American Tissue Culture Collection. The cells were nucleofected with Tax plasmid by using the Cell Line Nucleofector kit V and Nucleofector II (both from Lonza; the program T-014). The wild type or M22 mutant Tax derived from pX1 MT-M were flag-tagged and sub-cloned into the pIRES-neo3 vector (Invitrogen). The nucleofected cells were subjected to qRT-PCR for M-Sec or Tax mRNA. Jurkat reporter cells were also used in the co-culture assay (see below).

### Inhibitors

The M-Sec inhibitor (MSec-i) was identified by an affinity-based chemical array screening [8]. In brief, compounds were arrayed onto the photoaffinity linker-coated slides, which were incubated with the lysates of 293T cells expressing either DsRed or DsRed-M-Sec fusion proteins. The fluorescence signals were quantified, and the identified MSec-i was synthesized at Pharmeks (Moscow, Russia). BQU57 (Ral inhibitor), ZCL278 (Cdc42 inhibitor), and NSC23766 (Rac1 inhibitor) [26,27] were purchased from Sigma-Aldrich. MSec-i, BQU57, and ZCL278 were dissolved in DMSO, and NSC23766 was dissolved in H_2_O. These inhibitors were added to cultures at a final concentration of 10 μM (0.1% v/v), and the same volume of DMSO or H_2_O was added as a vehicle control.

### qRT-PCR for M-Sec- and Tax mRNA

The expression of M-Sec- or Tax mRNA was quantified using qRT-PCR and a 7500 Fast Real-Time PCR System (Applied Biosystems). GAPDH mRNA was also quantified as an internal control. The assay was performed in triplicate, and the levels of M-Sec- or Tax mRNA were calculated using the ΔΔCt method. The primer pairs were as follows: 5’-CGACACCTACATGCTG-3’ and 5’-CGAGCCCCATACCCTG-3’ (M-Sec), 5’- CCGGCGCTGCTCTCATCCCGGT-3’ and 5’- GGCCGAACATAGTCCCCCAGAG-3’ (Tax) [46], and 5’-ACCCACTCCTCCACCTTTGA-3’ and 5’-CTGTTGCTGTAGCCAAATTCGTT-3’ (GAPDH). In experiments in which Tax plasmid was used, Tax mRNA was quantified using another primer pair [47]: 5’-GGAACGGTGTCAGGATTCAAG-3’ and 5’-AGCGGCTGTACACCAGAAATG-3’.

### qPCR for provirus

The number of proviral copies was quantified using qPCR and a 7500 Fast Real-Time PCR system, as described previously [20]. The pX region of the provirus was amplified using the following primers and probe: 5’-CGGATACCCAGTCTACGTGTT-3’ (forward), 5’-CAGTAGGGCGTGACGATGTA-3’ (reverse), and 5’-CTGTGTACAAGGCGACTGGTGC C-3’ (probe) [47]. Human RNase P, a single copy gene, was also amplified as an internal control [20,47], using primers and probes purchased from Applied Biosystems. When humanized mouse tissues were analyzed, the human ribosomal protein S19 (RPS19) gene was used as an internal control [48], along with the following primers and probe: 5’-GGAACGGTGTCAGGATTCAAG-3’ (forward), 5’-AGCGGCTGTACACCAGAAATG-3’ (reverse), and 5’- TCTGACTGCTCTGGGCGCTAGTCCC- 3’ (probe). The assay was performed in triplicate, and the copy number of the pX region in each sample was determined relative to that in the reference TL-Om1 cells [20,47,49]. The number of proviral copies of samples was calculated as ([2 x copy of pX]/[copy of RNase P or RPS19]) x 100, and expressed as copies per 100 cells.

### Western blotting for M-Sec and Tax

Western blotting was performed as described previously [8,9]. The antibodies used were as follows: anti-M-Sec (F-6; Santa Cruz Biotechnology), anti-Tax (Lt-4) [50], and anti-α-tubulin (DM1A; Santa Cruz Biotechnology). Detection was performed with HRP-labeled secondary antibodies (GE Healthcare), Immunostar LD Western blotting detection reagent (Wako, Japan), and an image analyzer (LAS-3000; FujiFilm). Macrophages obtained by culturing monocytes of healthy volunteers in the presence of the cytokine M-CSF [8] were analyzed as a reference.

### Co-culture using cell lines

Jurkat cells carrying the firefly luciferase gene under the control of the HTLV-1 LTR promoter [51] were used as target cells. The cells were also transfected with the pRL-SV40 control Renilla luciferase plasmid (Promega). MT-2 or SLB-1 cells were used as infected cells. These cells were suspended in RPMI 1640 containing 10% FCS, mixed at a ratio of 1:1, seeded into 24-well plates (total 1 x 10^5^ cells per well), and cultured for 16 h. Luciferase activity was measured using a dual-luciferase reporter assay system and a Glomax 96 microplate luminometer (Promega), and firefly luciferase activity was normalized to control Renilla luciferase activity in the same sample. In an assay in which the effect of chemical inhibitors was tested, MT-2- or SLB-1 cells were pre-treated with each inhibitor for 48 h and used for the co-culture in which fresh antibodies were re-added. In a selected assay, MT-2- or SLB-1 cells were pre-treated with 20 μg/mL of anti-gp46 Env neutralizing antibody LAT-27 [24] for 1 h and used for the co-culture in which fresh antibodies were re-added.

### Co-culture using CD4^+^ T cells of HTLV-1 carriers

CD3^+^CD4^+^CADM1^+^ cells were sorted from PBMCs of HTLV-1 carriers as infected cells. Carriers with detectable levels of M-Sec expression in CD4^+^ T cells after *ex vivo* culture were selected for this assay. CD3^+^CD4^+^ cells were also sorted from the PBMCs of HTLV-1^-^ individuals as target cells. They were mixed at a ratio of 1:10 to 1:30, seeded into 96-well plates (3,000–5,000 cells/well for CD3^+^CD4^+^CADM1^+^ cells), and cultured with RPMI 1640/10% FCS/rhIL-2 for up to 4 days in the absence or presence of M-Sec- or Ral inhibitor. The genomic DNA of the cells in the co-culture was isolated and analyzed for proviral copies, as described above.

### Mouse model of HTLV-1 infection

Humanized mice were prepared by transplanting human hematopoietic stem cells (HSCs) into immunodeficient mice [20]. Immunodeficient NOD/SCID Jak3^null^ (NOJ) mice [52] were obtained from Kyudo (Saga, Japan). Human CD133^+^ HSCs [53] were enriched from cord blood (the Japanese Red Cross Cord Blood Bank, Tokyo, Japan), using the MicroBead kit (Miltenyi Biotec). Their purity was routinely > 90%. HSCs were intrahepatically transplanted into newborn NOJ mice (1 × 10^5^ cells/mouse). After 3 months, the number of human cells was measured using AccuCheck counting beads (Thermo Fisher). Human CD45^+^ cells in the peripheral blood are usually > 40%, in which human CD4^+^ T cells are > 20%. Mice with similar numbers of human cells were used for the inoculation of MT-2 cells.

The control or M-Sec knockdown MT-2 cells were irradiated using an MBR1505R2 X-ray cabinet system (70 Gy; Hitachi Power Solutions, Japan), and injected into the peritoneal cavity of the humanized mice (2 x 10^6^ cells/mouse). After 4 weeks, the cells in the peripheral blood, liver, spleen, and bone marrow were subjected to the isolation of genomic DNA, followed by proviral qPCR, as described above. Tissue samples were subjected to immunohistochemistry. The excised tissues were fixed in 10% phosphate-buffered formalin and embedded in paraffin. The paraffin blocks were cut into 3-μm-thick sections and mounted on silane-coated glass slides. The sections were stained with hematoxylin and eosin and processed for immunohistochemistry with anti-CD3 (2GV6; Roche) or anti-CD45 antibodies (NCL-L-LCA; Dako). For antigen retrieval, the sections were immersed in citrate buffer (pH 6.0) and heated for 10 min at 121°C. Specific antigen-antibody reactions were visualized using the EnVision+ system (Dako) for CD3 and the Mouse on Mouse kit (Vector Laboratories) for CD45. The number of positive cells in each section was estimated from those in areas with the highest cellularity of positive cells (no positive cells in the section = score 0, < 5 positive cells per high-power field (HPF) = score 1, < 50 positive cells per HPF = score 2, < 500 positive cells per HPF = score 3, and ≥ 500 positive cells per HPF = score 4).

### Cell migration

The migration of MT-2 cells was measured using a transwell assay with 5-μm pore size inserts (Corning). The inserts were placed into 24-well plates containing 600 μL RPMI1640/10% FCS in the absence or presence of rhSDF-1 (BioLegend) at a final concentration of 20 or 100 ng/mL. Then, cells were added to the inserts (2.5 x 10^5^ cells in 100 μL RPMI1640/10% FCS) and incubated at 37°C for 24 h. The number of cells that migrated through the inserts was enumerated using the trypan blue dye exclusion method.

### Membrane protrusion and cell morphology

Membrane protrusions were assessed using immunofluorescence, as described previously [8,9]. Cells were seeded onto BioCoat Poly-D-Lysine culture slides (Corning) and incubated for 30 min at 37°C to allow for adherence to the glass surface. The cells were then fixed in 4% paraformaldehyde, permeabilized with 0.1% Triton X-100, and incubated with phalloidin conjugated to AlexaFluor488 and DAPI (both from Molecular Probes) to visualize F-actin and nuclei, respectively. Signals were visualized using an FV1200 confocal laser-scanning microscope (Olympus), and image processing was performed using the FV Viewer ver. 4.1 soft ware (Olympus). If a cell had one or more F-actin^+^ protrusions longer than approximately 2 μm, it was considered protrusion^+^. The cell surface area, height, and diameter were also quantified using ImageJ 1.52n software (NIH) [9].

### Analyses of Gag

The intracellular distribution of Gag was visualized using immunofluorescence. In brief, cells were fixed, permeabilized, and stained with anti-p24 Gag antibodies (6G9; Santa Cruz Biotechnology) followed by anti-mouse IgG-AlexaFluor568 or anti-mouse IgG-AlexaFluor633 (both from Molecular Probes). The nuclei were stained with DAPI. Signals were visualized using an FV12 00 or FV3000 confocal laser-scanning microscope (Olympus), and image proce ssing was performed using FV Viewer ver. 4.1 or FV31S-SW software (both from Olympus). The amount of intracellular Gag was quantified using flow cytometry. The antibodies used were anti-p24 Gag (6G9) and APC-anti-mouse IgG (Molecular Probes).

### Statistics

Differences between groups were determined using the Student’s *t*-test. For multiple compariso ns, a two-way ANOVA with Sidak’s multiple comparisons test was used. *p* < 0.05 was considered significant. These analyses were performed using Prism software (GraphPad Software).

## Supporting information

S1 Table Provirus, Tax mRNA, and M-Sec mRNA in human CD3^+^ cells of MT-2-inoculated humanized mice. (PPTX)

S1 Fig KK-1 cells expressing GFP in a Tax-dependent manner. (related to Fig 2C). The reporter cassette expressing GFP under the control of the Tax-responsive element is schematically shown in the upper panel [18], and an example of GFP expression in KK-1 cells carrying the reporter cassette is shown in the lower panel.
(PDF)

S2 Fig Loss of ability of the Tax M22 mutant to activate the NF-κB promoter. (related to Fig 3B). To confirm that the Tax mutant M22 used in Fig 3B does not activate the NF-κB promoter [21], a co-transfection experiment using 293A cells and LipofectAMINE3000 (Invitrogen) was performed. The plasmids used were as follows: the empty vector, Tax expression plasmid (the wild type or M22), firefly luciferase reporter plasmid (NF-κB-Luc; a gift from H. Iha, Oita University, Japan), and pRL-SV40 control Renilla luciferase plasmid (Promega). Luciferase activities were measured as described in Materials and Methods section, and are shown by setting the value of the empty vector/luciferase plasmid-transfected cells as 1 (n = 3). *p < 0.05. *n*.*s*., not significant.
(PDF)

S3 Fig Establishment of M-Sec knockdown MT-2 or SLB-1 cells. The control (Cr)- or M-Sec knockdown (MSec-KD) MT-2- or SLB-1 cells were analyzed for the expression of M-Sec protein by using western blotting. α-tubulin blot is the loading control. The control cells pre-treated with M-Sec inhibitor (MSec-i; its structure is shown in upper right) for 48 h were also added (Cr/MSec-i). The M-Sec inhibitor does not affect the protein level of M-Sec. Bulk cultures of puromycin-selected M-Sec knockdown cells without cloning were used throughout this study to exclude the possibility of clonal variation.
(PDF)

S4 Fig Expression of M-Sec in CD3^+^CD4^+^CADM1^+^ cells. (**A**) The CD3^+^CD4^+^CADM1^+^ cells in the live cell gate were sorted from PBMCs of HTLV-1 carriers. The profile of an HTLV-1 carrier is shown as an example. (**B**) The CD3^+^CD4^+^CADM1^+^ cells sorted from PBMCs of an HTLV-1 carrier were cultured for 3 days, and analyzed for M-Sec (green) and CD3 (red). The nuclei were also stained with DAPI (blue). Monocytes were added as a positive control for M-Sec. The antibodies used for staining were as follows: anti-M-Sec (F-6; Santa Cruz Biotechnology), and anti-CD3 (CD3-12; Abcam). Scale bar: 10 μm. (**C**) The cells were analyzed as in (**B**), and the percentages of CD3^+^CD4^+^CADM1^+^ cells or monocytes expressing M-Sec at a detectable level are shown (15 cells for each). The typical signal of monocytes was defined as “bright”.
(PDF)

S5 Fig Effect of M-Sec inhibition on viral infection in co-culture with primary cells. (related to Fig 5). The original data in Fig 5 are shown. The Y-axis represents the number of proviral copies per 100 cells in the co-culture.
(PDF)

S6 Fig Effect of M-Sec knockdown and inhibitor treatment on cell proliferation. (related to Figs 4 and 5). (**A**) The control (Cr)- or M-Sec knockdown (MSec-KD) MT-2- or SLB-1 cells were cultured for the indicated periods, and cell number was counted using the trypan blue dye exclusion method (n = 3). The control cells were also cultured in the presence of M-Sec inhibitor (Cr/MSec-i). (**B**) Jurkat cells were cultured in the absence (None) or presence of M-Sec inhibitor (MSec-i), and analyzed as described in (**A**). (**C**) The control (Cr) MT-2- or SLB-1 cells (left), or Jurkat cells (right) were cultured in the absence (None) or presence of Ral inhibitor (Ral-i), and analyzed as in (**A**). (**D**) The CD3^+^CD4^+^ T cells in the live cell gate were sorted from PBMCs of HTLV-1^-^ individuals, and cultured in the absence (None) or presence of M-Sec inhibitor (MSec-i) or Ral inhibitor (Ral-i) for the indicated periods, and cell numbers were counted as in (**A**). The results shown are the summary of cells obtained from three different individuals.
(PDF)

S7 Fig Number of MT-2 cells in the spleen of mice. (related to Fig 6A). (**A**) The un-humanized immunodeficient mice were inoculated intraperitoneally with un-irradiated control (Cr)- or M-Sec knockdown (MSec-KD) MT-2 cells (1 x 10^7^ cells/mouse). To monitor MT-2 cells, cells in the spleen were analyzed on days 3, 7, and 14 for proviral copies by using qPCR (PCR) or flow cytometry (FCM). In flow cytometry, the inoculated MT-2 were identified as cells positive for both GFP and human CD25. The numbers of MT-2 are shown by setting the value of control MT-2-inoculated spleen on day 3 as 1. *n*.*s*., not significant. (**B**) The un-humanized immunodeficient mice were inoculated intraperitoneally with un-irradiated control- or M-Sec knockdown (MSec-KD) MT-2 cells (5 x 10^7^ cells/mouse), and analyzed on day 21 for proviral copies by using qPCR. The numbers of MT-2 are shown by setting the mean value of control MT-2-inoculated spleen as 1.
(PDF)

S8 Fig Proliferation of M-Sec knockdown MT-2 cells. To further confirm that M-Sec knockdown did not affect the proliferation of MT-2 cells (S6A Fig), a serial passage of MT-2 cells was also performed. First, the control (Cr) or M-Sec knockdown (MSec-KD) MT-2 cells were analyzed for the expression of M-Sec protein by using western blotting (upper left). The results of densitometric analysis of the bands are shown. They were then serially passaged. In each passage, the cells were cultured for 3 days, and cell numbers were enumerated using the trypan blue dye exclusion method (lower panels). After 4th passage, the cells were re-analyzed for the expression M-Sec protein by western blotting (upper right).
(PDF)

S9 Fig Proliferation of M-Sec knockdown RAW264 cells. The control (Cr)- or M-Sec knockdown (MSec-KD) RAW264 cells were provided by H. Ohno (RIKEN, Japan) [3]. The cells were seeded (0.5 x 10^5^ cells/well) and cultured with RPMI 1640/10% FCS for 3 days, and cell numbers were enumerated using the trypan blue dye exclusion method (n = 3). *n*.*s*., not significant.
(PDF)

S10 Fig Intracellular distribution of Gag in MT-2- and SLB-1 cells. The control MT-2 cells (upper panels) or SLB-1 cells (lower panels) were analyzed for Gag, Calnexin (as an endoplasmic reticulum marker), or GM130 (as a Golgi marker). The nuclei were also stained with DAPI. The antibodies used for staining were as follows: anti-Calnexin (C5C9; Cell Signaling Biotechnology), and GM130 (D6B1; Cell Signaling Biotechnology). Scale bar: 10 μm.
(PDF)

S11 Fig Serial Z-sections of large Gag clusters in MT-2 cells. (related to Fig 8). The control MT-2 cells were stained as in Fig 8, and serial Z-sections from the top to the bottom are shown (left). An overlay image of the serial Z-sections is also shown (right). A yellow arrowhead indicates a typical large Gag cluster, which is composed of many puncta. Scale bar: 10 μm.
(PDF)

S12 Fig Effect of M-Sec knockdown on the intracellular distribution of Gag in SLB-1 cells. (related to Fig 8). (**A**) The control (Cr)- or M-Sec knockdown (MSec-KD) SLB-1 cells were analyzed for Gag (red). The nuclei were also stained with DAPI (blue). Scale bar: 10 μm. (**B**) The cells were analyzed as in (**A**). Three different fields were randomly selected, and the percentages of large Gag cluster^+^ cells were quantified (upper). The numbers of small clusters of Gag per cell are also shown (lower, 16 cells for each). *p < 0.05.
(PDF)

S13 Fig Effect of M-Sec knockdown on viral infection in MT-2/Jurkat co-culture. (related to Fig 4A). Reporter Jurkat cells were co-cultured with control (Cr) or M-Sec knockdown (MSec-KD) MT-2 cells for 16 or 36 h. Luciferase activities are shown by setting the value of Jurkat alone as 1 (n = 3). *p < 0.05. *n*.*s*., not significant.
(PDF)

S1 Video Intracellular distribution of Gag in control MT-2 cells. The control MT-2 cells were analyzed for Gag (red). The nuclei were also stained with DAPI (blue).
(PPTX)

S2 Video Intracellular distribution of Gag in M-Sec knockdown MT-2 cells. M-Sec knockdown MT-2 cells were analyzed for Gag (red). The nuclei were also stained with DAPI (blue).
(PPTX)

## References

[1] Rustom A, Saffrich R, Markovic I, Walther P, Gerdes HH. Nanotubular highways for intercellular organelle transport. Science 2004;303: 1007–1010. doi: 10.1126/science.1093133 14963329

[2] Zurzolo C. Tunneling nanotubes: reshaping connectivity. Curr Opin Cell Biol 2021;71: 139–147. doi: 10.1016/j.ceb.2021.03.003 33866130

[3] Hase K, Kimura S, Takatsu H, Ohmae M, Kawano S, Kitamura H, et al. M-Sec promotes membrane nanotube formation by interacting with Ral and the exocyst complex. Nat Cell Biol 2009;11: 1427–1432. doi: 10.1038/ncb1990 19935652

[4] Kimura S, Yamashita M, Yamakami-Kimura M, Sato Y, Yamagata A, Kobashigawa Y, et al. Distinct roles for the N- and C-terminal regions of M-Sec in plasma membrane deformation during tunneling nanotube formation. Sci Rep 2016;6: 33548. doi: 10.1038/srep33548 27629377 PMC5024327

[5] Chen CC, Liu HP, Chao M, Liang Y, Tsang NM, Huang HY, et al. NF-κB-mediated transcriptional upregulation of TNFAIP2 by the Epstein-Barr virus oncoprotein, LMP1, promotes cell motility in nasopharyngeal carcinoma. Oncogene 2014;33: 3648–3659. doi: 10.1038/onc.2013.345 23975427

[6] Jia L, Zhou Z, Liang H, Wu J, Shi P, Li F, et al. KLF5 promotes breast cancer proliferation, migration and invasion in part by upregulating the transcription of TNFAIP2. Oncogene 2016;35: 2040–2051. doi: 10.1038/onc.2015.263 26189798

[7] Xie Y, Wang B. Downregulation of TNFAIP2 suppresses proliferation and metastasis in esophageal squamous cell carcinoma through activation of the Wnt/β-catenin signaling pathway. Oncol Rep 2017;37: 2920–2928. doi: 10.3892/or.2017.5557 28393234

[8] Hashimoto M, Bhuyan F, Hiyoshi M, Noyori O, Nasser H, Miyazaki M, et al. Potential role of the formation of tunneling nanotubes in HIV-1 spread in macrophages. J Immunol 2016;196: 1832–1841. doi: 10.4049/jimmunol.1500845 26773158

[9] Lotfi S, Nasser H, Noyori O, Hiyoshi M, Takeuchi H, Koyanagi Y, et al. M-Sec facilitates intercellular transmission of HIV-1 through multiple mechanisms. Retrovirology 2020;17: 20. doi: 10.1186/s12977-020-00528-y 32650782 PMC7350586

[10] Gessain A, Cassar O. Epidemiological aspects and world distribution of HTLV-1 infection. Front Microbiol 2012;3: 388. doi: 10.3389/fmicb.2012.00388 23162541 PMC3498738

[11] Schierhout G, McGregor S, Gessain A, Einsiedel L, Martinello M, Kaldor J. Association between HTLV-1 infection and adverse health outcomes: a systematic review and meta-analysis of epidemiological studies. Lancet Infect Dis 2020;20: 133–143. doi: 10.1016/S1473-3099(19)30402-5 31648940

[12] Igakura T, Stinchcombe JC, Goon PK, Taylor GP, Weber JN, Griffiths GM, et al. Spread of HTLV-I between lymphocytes by virus-induced polarization of the cytoskeleton. Science 2003;299: 1713–1716. doi: 10.1126/science.1080115 12589003

[13] Jones KS, Petrow-Sadowski C, Huang YK, Bertolette DC, Ruscetti FW. Cell-free HTLV-1 infects dendritic cells leading to transmission and transformation of CD4^+^ T cells. Nat Med 2008;14: 429–436. doi: 10.1038/nm1745 18376405

[14] Dutartre H, Clavière M, Journo C, Mahieux R. Cell-free versus cell-to-cell infection by human immunodeficiency virus type 1 and human T-lymphotropic virus type 1: exploring the link among viral source, viral trafficking, and viral replication. J Virol 2016;90: 7607–7617. doi: 10.1128/JVI.00407-16 27334587 PMC4988172

[15] Pais-Correia AM, Sachse M, Guadagnini S, Robbiati V, Lasserre R, Gessain A, et al. Biofilm-like extracellular viral assemblies mediate HTLV-1 cell-to-cell transmission at virological synapses. Nat Med. 2010;16: 83–89. doi: 10.1038/nm.2065 20023636

[16] Van Prooyen N, Gold H, Andresen V, Schwartz O, Jones K, Ruscetti F, et al. Human T-cell leukemia virus type 1 p8 protein increases cellular conduits and virus transmission. Proc Natl Acad Sci U S A 2010;107: 20738–20743. doi: 10.1073/pnas.1009635107 21076035 PMC2996430

[17] Kulkarni A, Taylor GP, Klose RJ, Schofield CJ, Bangham CR. Histone H2A monoubiquitylation and p38-MAPKs regulate immediate-early gene-like reactivation of latent retrovirus HTLV-1. JCI Insight 2018;3: e123196. doi: 10.1172/jci.insight.123196 30333309 PMC6237452

[18] Mahgoub M, Yasunaga JI, Iwami S, Nakaoka S, Koizumi Y, Shimura K, et al. Sporadic on/off switching of HTLV-1 Tax expression is crucial to maintain the whole population of virus-induced leukemic cells. Proc Natl Acad Sci U S A 2018;115: E1269–E1278. doi: 10.1073/pnas.1715724115 29358408 PMC5819419

[19] Miura M, Dey S, Ramanayake S, Singh A, Rueda DS, Bangham CRM. Kinetics of HTLV-1 reactivation from latency quantified by single-molecule RNA FISH and stochastic modelling. PLoS Pathog 2019;15: e1008164. doi: 10.1371/journal.ppat.1008164 31738810 PMC6886867

[20] Hiyoshi M, Okuma K, Tateyama S, Takizawa K, Saito M, Kuramitsu M, et al. Furin-dependent CCL17-fused recombinant toxin controls HTLV-1 infection by targeting and eliminating infected CCR4-expressing cells in vitro and in vivo. Retrovirology 2015;12: 73. doi: 10.1186/s12977-015-0199-8 26289727 PMC4545545

[21] Geleziunas R, Ferrell S, Lin X, Mu Y, Cunningham ET, Grant M, et al. Human T-cell leukemia virus type 1 Tax induction of NF-κB involves activation of the IκB kinase alpha (IKKα) and IKKβ cellular kinases. Mol Cell Biol. 1998;18: 5157–5165. doi: 10.1128/MCB.18.9.5157 9710600 PMC109101

[22] Pergu R, Dagar S, Kumar H, Kumar R, Bhattacharya J, Mylavarapu SVS. The chaperone ERp29 is required for tunneling nanotube formation by stabilizing MSec. J Biol Chem 2019;294: 7177–7193. doi: 10.1074/jbc.RA118.005659 30877198 PMC6509506

[23] Souriant S, Balboa L, Dupont M, Pingris K, Kviatcovsky D, Cougoule C, et al. Tuberculosis exacerbates HIV-1 infection through IL-10/STAT3-dependent tunneling nanotube formation in macrophages. Cell Rep 2019;26: 3586–3599.e7. doi: 10.1016/j.celrep.2019.02.091 30917314 PMC6733268

[24] Tanaka Y, Takahashi Y, Tanaka R, Kodama A, Fujii H, Hasegawa A, et al. Elimination of human T cell leukemia virus type-1-infected cells by neutralizing and antibody-dependent cellular cytotoxicity-inducing antibodies against human T cell leukemia virus type-1 envelope gp46. AIDS Res Hum Retroviruses 2014;30: 542–552. doi: 10.1089/aid.2013.0214 24524420

[25] Lanzetti L. Actin in membrane trafficking. Curr Opin Cell Biol. 2007;19: 453–458. doi: 10.1016/j.ceb.2007.04.017 17616384

[26] Yan C, Liu D, Li L, Wempe MF, Guin S, Khanna M, et al. Discovery and characterization of small molecules that target the GTPase Ral. Nature 2014;515: 443–447. doi: 10.1038/nature13713 25219851 PMC4351747

[27] Friesland A, Zhao Y, Chen YH, Wang L, Zhou H, Lu Q. Small molecule targeting Cdc42-intersectin interaction disrupts Golgi organization and suppresses cell motility. Proc Natl Acad Sci U S A 2013;110: 1261–1266. doi: 10.1073/pnas.1116051110 23284167 PMC3557054

[28] Manivannan K, Rowan AG, Tanaka Y, Taylor GP, Bangham CR. CADM1/TSLC1 identifies HTLV-1-infected cells and determines their susceptibility to CTL-mediated lysis. PLoS Pathog 2016;12: e1005560. doi: 10.1371/journal.ppat.1005560 27105228 PMC4841533

[29] Villaudy J, Wencker M, Gadot N, Gillet NA, Scoazec JY, Gazzolo L, et al. HTLV-1 propels thymic human T cell development in "human immune system" Rag2-/- gamma c-/- mice. PLoS Pathog 2011;7: e1002231. doi: 10.1371/journal.ppat.1002231 21909275 PMC3164654

[30] Percher F, Curis C, Pérès E, Artesi M, Rosewick N, Jeannin P, et al. HTLV-1-induced leukotriene B4 secretion by T cells promotes T cell recruitment and virus propagation. Nat Commun 2017;8: 15890. doi: 10.1038/ncomms15890 28639618 PMC5489682

[31] Le Blanc I, Blot V, Bouchaert I, Salamero J, Goud B, Rosenberg AR, et al. Intracellular distribution of human T-cell leukemia virus type 1 Gag proteins is independent of interaction with intracellular membranes. J Virol 2002;76: 905–911. doi: 10.1128/jvi.76.2.905-911.2002 11752179 PMC136804

[32] Inlora J, Chukkapalli V, Derse D, Ono A. Gag localization and virus-like particle release mediated by the matrix domain of human T-lymphotropic virus type 1 Gag are less dependent on phosphatidylinositol-(4,5)-bisphosphate than those mediated by the matrix domain of HIV-1 Gag. J Virol 2011; 85: 3802–3810. doi: 10.1128/JVI.02383-10 21289126 PMC3126146

[33] Inlora J, Collins DR, Trubin ME, Chung JY, Ono A. Membrane binding and subcellular localization of retroviral Gag proteins are differentially regulated by MA interactions with phosphatidylinositol-(4,5)-bisphosphate and RNA. mBi. 2014; 5: e02202. 10.1128/mBio.02202-14 PMC4324246 25491356

[34] Martin JL, Mendonça LM, Marusinec R, Zuczek J, Angert I, Blower RJ, et al. Critical role of the human T-cell leukemia virus type 1 capsid N-terminal domain for Gag-Gag interactions and virus particle assembly. J Virol 2018;92: e00333–18. doi: 10.1128/JVI.00333-18 29695435 PMC6026748

[35] Eichorst JP, Chen Y, Mueller JD, Mansky LM. Distinct pathway of human T-cell leukemia virus type 1 Gag punctum biogenesis provides new insights into enveloped virus assembly. mBio 2018;9: e00758–18. doi: 10.1128/mBio.00758-18 30181245 PMC6123448

[36] Chan JK, Greene WC. Dynamic roles for NF-κB in HTLV-I and HIV-1 retroviral pathogenesis. Immunol Rev 2012;246: 286–310. doi: 10.1111/j.1600-065X.2012.01094.x 22435562

[37] Heusinger E, Kirchhoff F. Primate lentiviruses modulate NF-κB activity by multiple mechanisms to fine-tune viral and cellular gene expression. Front Microbiol 2017;8: 198. doi: 10.3389/fmicb.2017.00198 28261165 PMC5306280

[38] Bangham CRM, Miura M, Kulkarni A, Matsuoka M. Regulation of latency in the human T cell leukemia virus, HTLV-1. Annu Rev Virol 2019;6: 365–385. doi: 10.1146/annurev-virology-092818-015501 31283437

[39] Gross C, Wiesmann V, Millen S, Kalmer M, Wittenberg T, Gettemans J, et al. The Tax-inducible actin-bundling protein fascin is crucial for release and cell-to-cell transmission of human T-cell leukemia virus type 1 (HTLV-1). PLoS Pathog 2016;12: e1005916. doi: 10.1371/journal.ppat.1005916 27776189 PMC5077169

[40] Varrin-Doyer M, Nicolle A, Marignier R, Cavagna S, Benetollo C, Wattel E, et al. Human T lymphotropic virus type 1 increases T lymphocyte migration by recruiting the cytoskeleton organizer CRMP2. J Immunol 2012;188: 1222–1233. doi: 10.4049/jimmunol.1101562 22227566

[41] Chevalier SA, Turpin J, Cachat A, Afonso PV, Gessain A, Brady JN, et al. Gem-induced cytoskeleton remodeling increases cellular migration of HTLV-1-infected cells, formation of infected-to-target T-cell conjugates and viral transmission. PLoS Pathog 2014;10: e1003917. doi: 10.1371/journal.ppat.1003917 24586148 PMC3937318

[42] Jensen LJ, Kuhn M, Stark M, Chaffron S, Creevey C, Muller J, et al. STRING 8—a global view on proteins and their functional interactions in 630 organisms. Nucleic Acids Res 2009; 37: D412–416. doi: 10.1093/nar/gkn760 18940858 PMC2686466

[43] Du Bois I, Marsico A, Bertrams W, Schweiger MR, Caffrey BE, Sittka-Stark A, et al. Genome-wide chromatin profiling of Legionella pneumophila-infected human macrophages reveals activation of the probacterial host factor TNFAIP2. J Infect Dis 2016; 214: 454–463. doi: 10.1093/infdis/jiw171 27130431

[44] Furuta R, Yasunaga JI, Miura M, Sugata K, Saito A, Akari H, et al. Human T-cell leukemia virus type 1 infects multiple lineage hematopoietic cells *in vivo*. PLoS Pathog 2017;13: e1006722. doi: 10.1371/journal.ppat.1006722 29186194 PMC5724899

[45] Mitchell MS, Bodine ET, Hill S, Princler G, Lloyd P, Mitsuya H, et al. Phenotypic and genotypic comparisons of human T-cell leukemia virus type 1 reverse transcriptases from infected T-cell lines and patient samples. J Virol 2007;81: 4422–4428. doi: 10.1128/JVI.02660-06 17287279 PMC1900182

[46] Satou Y, Yasunaga J, Yoshida M, Matsuoka M. HTLV-I basic leucine zipper factor gene mRNA supports proliferation of adult T cell leukemia cells. Proc Natl Acad Sci U S A 2006;103: 720–725. doi: 10.1073/pnas.0507631103 16407133 PMC1334651

[47] Watanabe M, Ohsugi T, Shoda M, Ishida T, Aizawa S, Maruyama-Nagai M, et al. Dual targeting of transformed and untransformed HTLV-1-infected T cells by DHMEQ, a potent and selective inhibitor of NF-κB, as a strategy for chemoprevention and therapy of adult T-cell leukemia. Blood 2005;106: 2462–2471. doi: 10.1182/blood-2004-09-3646 15956280

[48] Kuramitsu M, Sato-Otsubo A, Morio T, Takagi M, Toki T, Terui K, et al. Extensive gene deletions in Japanese patients with Diamond-Blackfan anemia. Blood 2012;119: 2376–2384. doi: 10.1182/blood-2011-07-368662 22262766

[49] Kuramitsu M, Okuma K, Yamagishi M, Yamochi T, Firouzi S, Momose H, et al. Identification of TL-Om1, an adult T-cell leukemia (ATL) cell line, as reference material for quantitative PCR for human T-lymphotropic virus 1. J Clin Microbiol 2015;53: 587–596. doi: 10.1128/JCM.02254-14 25502533 PMC4298509

[50] Lee B, Tanaka Y, Tozawa H. Monoclonal antibody defining tax protein of human T-cell leukemia virus type-I. Tohoku J Exp Med 1989;157: 1–11. doi: 10.1620/tjem.157.1 2711372

[51] Fujisawa J, Seiki M, Kiyokawa T, Yoshida M. Functional activation of the long terminal repeat of human T-cell leukemia virus type I by a trans-acting factor. Proc Natl Acad Sci U S A 1985;82: 2277–2281. doi: 10.1073/pnas.82.8.2277 2986109 PMC397540

[52] Okada S, Harada H, Ito T, Saito T, Suzu S. Early development of human hematopoietic and acquired immune systems in new born NOD/Scid/Jak3null mice intrahepatic engrafted with cord blood-derived CD34^+^ cells. Int J Hematol 2008;88: 476–482. doi: 10.1007/s12185-008-0215-z 19039627

[53] Iwabuchi R, Ikeno S, Kobayashi-Ishihara M, Takeyama H, Ato M, Tsunetsugu-Yokota Y, et al. Introduction of human Flt3-L and GM-CSF into humanized mice enhances the reconstitution and maturation of myeloid dendritic cells and the development of Foxp3+CD4+ T cells. Front Immunol 2018;9: 1042. doi: 10.3389/fimmu.2018.01042 29892279 PMC5985304

