# Supplementary material for: M-Sec induced by HTLV-1 mediates an efficient viral transmission
Source: PLoS Pathog. 2021 Nov 29;17(11):e1010126. doi: 10.1371/journal.ppat.1010126 (PMC8659635; doi:10.1371/journal.ppat.1010126)
Supplement: S1 Table — (PPTX) [file ppat.1010126.s001.pptx]

## Slide 1
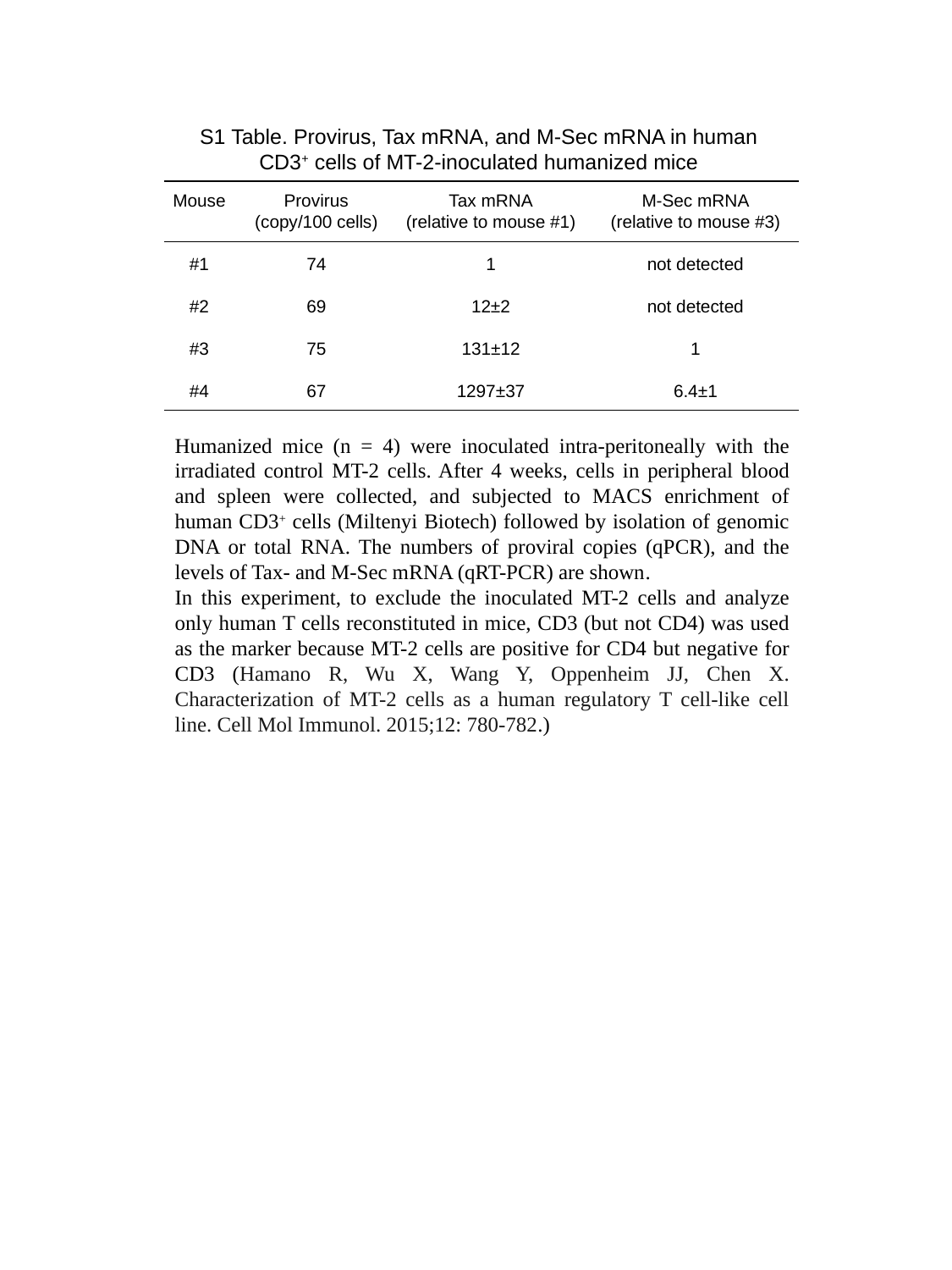

S1 Table. Provirus, Tax mRNA, and M-Sec mRNA in human CD3+ cells of MT-2-inoculated humanized mice
Mouse
#1
#2
#3
#4
Provirus
(copy/100 cells)
74
69
75
67
Tax mRNA
(relative to mouse #1)
1
12±2
131±12
1297±37
M-Sec mRNA
(relative to mouse #3)
not detected
not detected
1
6.4±1
Humanized mice (n = 4) were inoculated intra-peritoneally with the irradiated control MT-2 cells. After 4 weeks, cells in peripheral blood and spleen were collected, and subjected to MACS enrichment of human CD3+ cells (Miltenyi Biotech) followed by isolation of genomic DNA or total RNA. The numbers of proviral copies (qPCR), and the levels of Tax- and M-Sec mRNA (qRT-PCR) are shown.
In this experiment, to exclude the inoculated MT-2 cells and analyze only human T cells reconstituted in mice, CD3 (but not CD4) was used as the marker because MT-2 cells are positive for CD4 but negative for CD3 (Hamano R, Wu X, Wang Y, Oppenheim JJ, Chen X. Characterization of MT-2 cells as a human regulatory T cell-like cell line. Cell Mol Immunol. 2015;12: 780-782.)
