# Supplementary figures and images for: M-Sec induced by HTLV-1 mediates an efficient viral transmission
Source: PLoS Pathog. 2021 Nov 29;17(11):e1010126. doi: 10.1371/journal.ppat.1010126 (PMC8659635; doi:10.1371/journal.ppat.1010126)

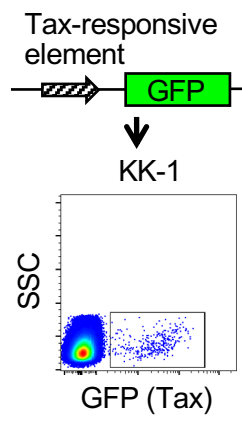

S1 Fig  
(related to Fig 2C)

Supplement: S1 Fig — (related to Fig 2C). The reporter cassette expressing GFP under the control of the Tax-responsive element is schematically shown in the upper panel [18], and an example of GFP expression in KK-1 cells carrying the reporter cassette is shown in the lower panel. (PDF) [file ppat.1010126.s002.pdf]

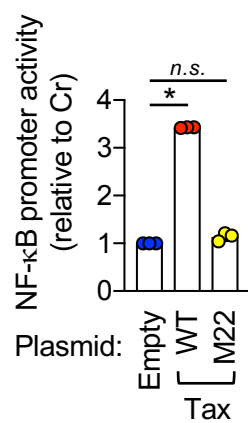

S2 Fig  
(related to Fig 3B)

Supplement: S2 Fig — (related to Fig 3B). To confirm that the Tax mutant M22 used in Fig 3B does not activate the NF-κB promoter [21], a co-transfection experiment using 293A cells and LipofectAMINE3000 (Invitrogen) was performed. The plasmids used were as follows: the empty vector, Tax expression plasmid (the wild type or M22), firefly luciferase reporter plasmid (NF-κB-Luc; a gift from H. Iha, Oita University, Japan), and pRL-SV40 control Renilla luciferase plasmid (Promega). Luciferase activities were measured as described in Materials and Methods section, and are shown by setting the value of the empty vector/luciferase plasmid-transfected cells as 1 (n = 3). *p < 0.05. n.s., not significant. (PDF) [file ppat.1010126.s003.pdf]

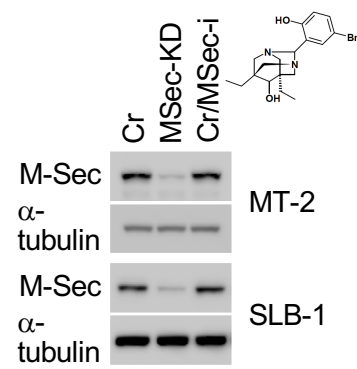

S3 Fig

Supplement: S3 Fig — The control (Cr)- or M-Sec knockdown (MSec-KD) MT-2- or SLB-1 cells were analyzed for the expression of M-Sec protein by using western blotting. α-tubulin blot is the loading control. The control cells pre-treated with M-Sec inhibitor (MSec-i; its structure is shown in upper right) for 48 h were also added (Cr/MSec-i). The M-Sec inhibitor does not affect the protein level of M-Sec. Bulk cultures of puromycin-selected M-Sec knockdown cells without cloning were used throughout this study to exclude the possibility of clonal variation. (PDF) [file ppat.1010126.s004.pdf]

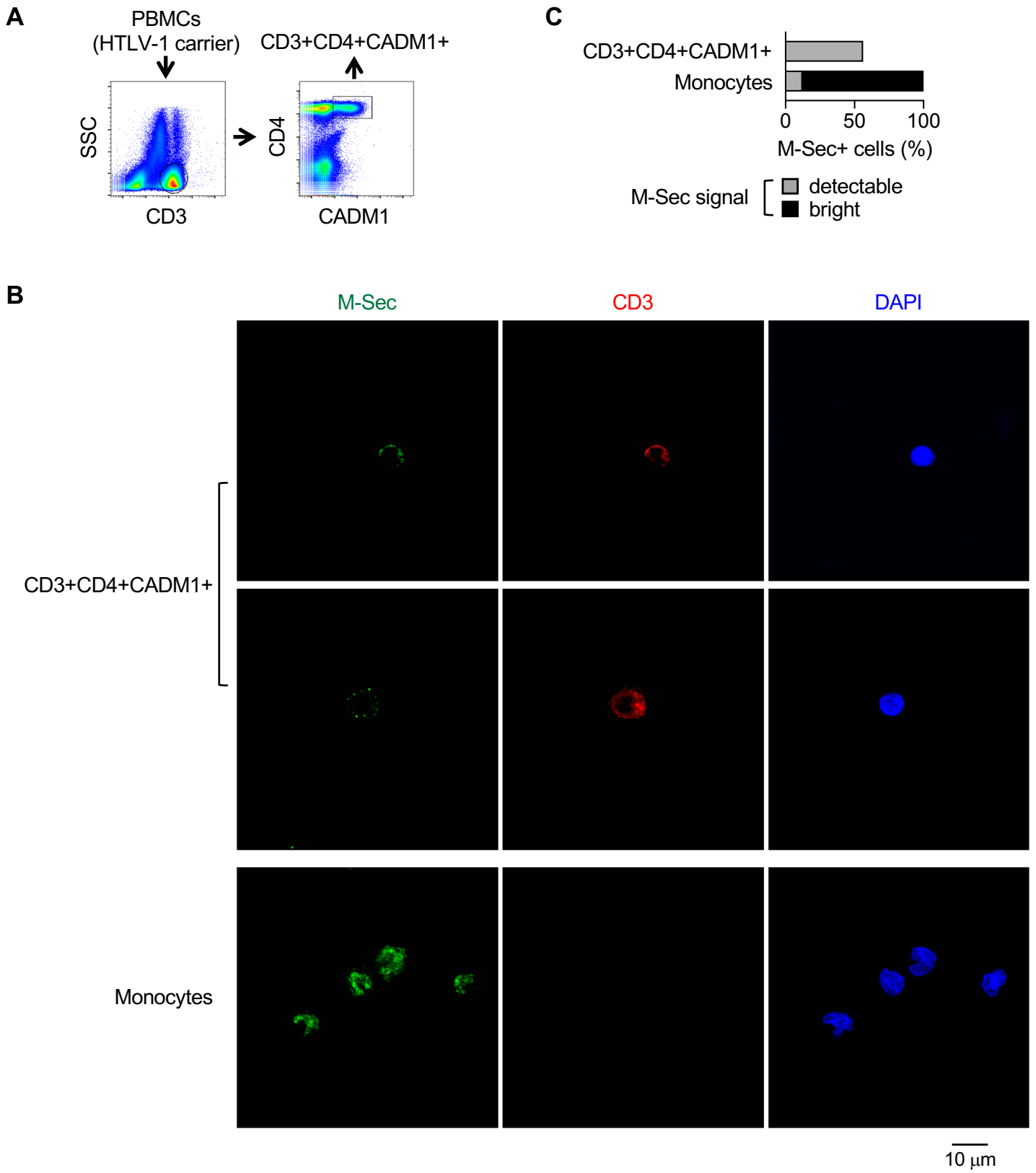

S4 Fig

Supplement: S4 Fig — (A) The CD3+CD4+CADM1+ cells in the live cell gate were sorted from PBMCs of HTLV-1 carriers. The profile of an HTLV-1 carrier is shown as an example. (B) The CD3+CD4+CADM1+ cells sorted from PBMCs of an HTLV-1 carrier were cultured for 3 days, and analyzed for M-Sec (green) and CD3 (red). The nuclei were also stained with DAPI (blue). Monocytes were added as a positive control for M-Sec. The antibodies used for staining were as follows: anti-M-Sec (F-6; Santa Cruz Biotechnology), and anti-CD3 (CD3-12; Abcam). Scale bar: 10 μm. (C) The cells were analyzed as in (B), and the percentages of CD3+CD4+CADM1+ cells or monocytes expressing M-Sec at a detectable level are shown (15 cells for each). The typical signal of monocytes was defined as “bright”. (PDF) [file ppat.1010126.s005.pdf]

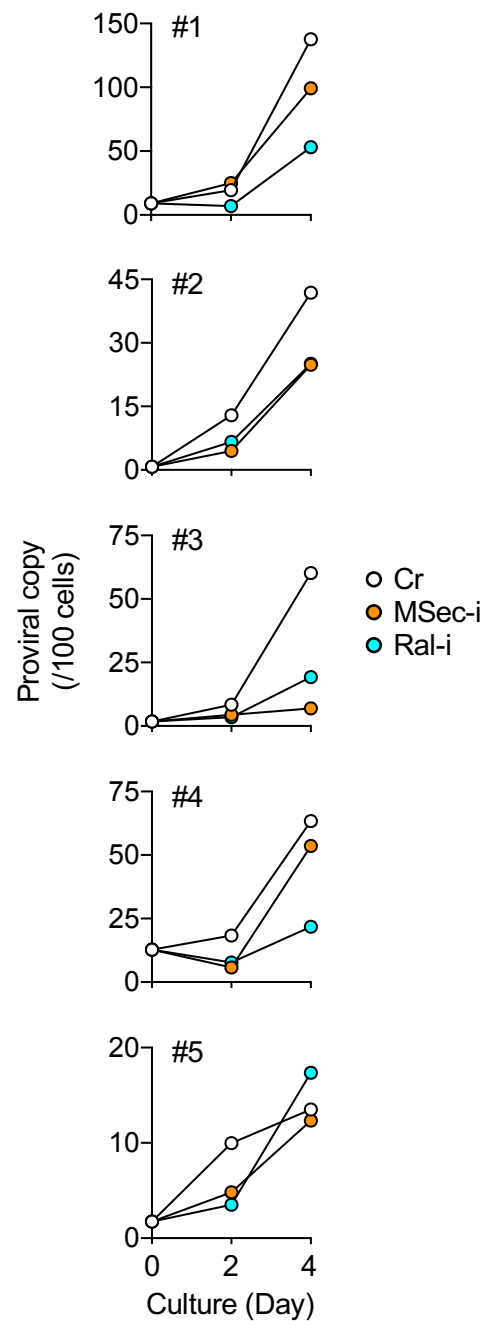

S5 Fig  
(related to Fig 5)

Supplement: S5 Fig — (related to Fig 5). The original data in Fig 5 are shown. The Y-axis represents the number of proviral copies per 100 cells in the co-culture. (PDF) [file ppat.1010126.s006.pdf]

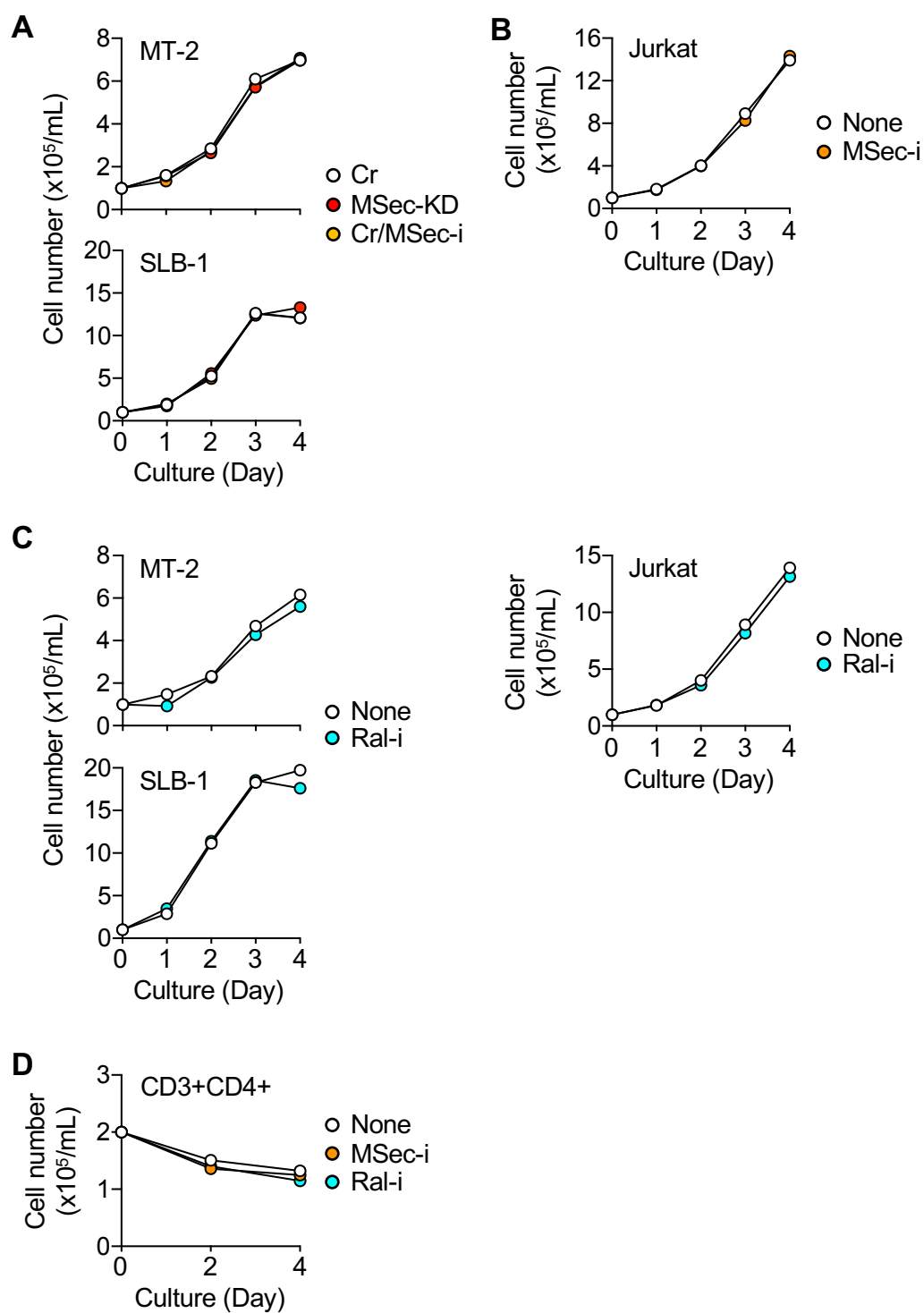

S6 Fig  
(related to Fig 4 and 5)

Supplement: S6 Fig — (related to Figs 4 and 5). (A) The control (Cr)- or M-Sec knockdown (MSec-KD) MT-2- or SLB-1 cells were cultured for the indicated periods, and cell number was counted using the trypan blue dye exclusion method (n = 3). The control cells were also cultured in the presence of M-Sec inhibitor (Cr/MSec-i). (B) Jurkat cells were cultured in the absence (None) or presence of M-Sec inhibitor (MSec-i), and analyzed as described in (A). (C) The control (Cr) MT-2- or SLB-1 cells (left), or Jurkat cells (right) were cultured in the absence (None) or presence of Ral inhibitor (Ral-i), and analyzed as in (A). (D) The CD3+CD4+ T cells in the live cell gate were sorted from PBMCs of HTLV-1- individuals, and cultured in the absence (None) or presence of M-Sec inhibitor (MSec-i) or Ral inhibitor (Ral-i) for the indicated periods, and cell numbers were counted as in (A). The results shown are the summary of cells obtained from three different individuals. (PDF) [file ppat.1010126.s007.pdf]

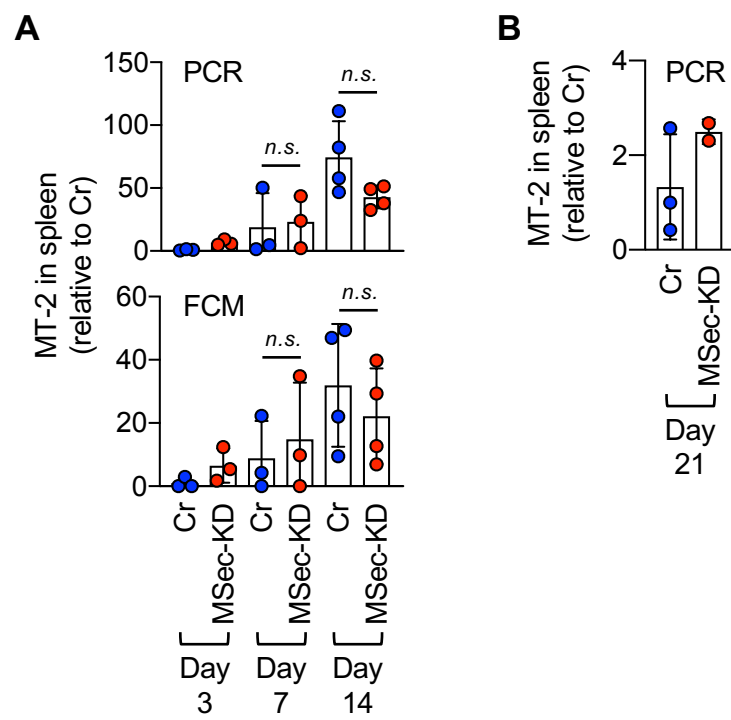

S7 Fig  
(related to Fig 6A)

Supplement: S7 Fig — (related to Fig 6A). (A) The un-humanized immunodeficient mice were inoculated intraperitoneally with un-irradiated control (Cr)- or M-Sec knockdown (MSec-KD) MT-2 cells (1 x 10^7^ cells/mouse). To monitor MT-2 cells, cells in the spleen were analyzed on days 3, 7, and 14 for proviral copies by using qPCR (PCR) or flow cytometry (FCM). In flow cytometry, the inoculated MT-2 were identified as cells positive for both GFP and human CD25. The numbers of MT-2 are shown by setting the value of control MT-2-inoculated spleen on day 3 as 1. n.s., not significant. (B) The un-humanized immunodeficient mice were inoculated intraperitoneally with un-irradiated control- or M-Sec knockdown (MSec-KD) MT-2 cells (5 x 10^7^ cells/mouse), and analyzed on day 21 for proviral copies by using qPCR. The numbers of MT-2 are shown by setting the mean value of control MT-2-inoculated spleen as 1. (PDF) [file ppat.1010126.s008.pdf]

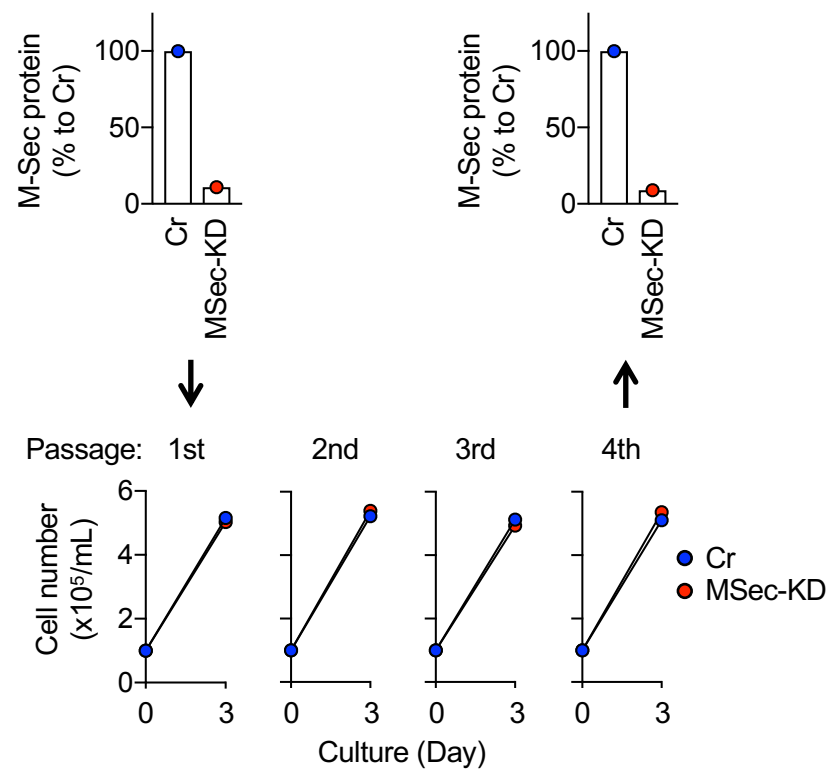

S8 Fig

Supplement: S8 Fig — To further confirm that M-Sec knockdown did not affect the proliferation of MT-2 cells (S6A Fig), a serial passage of MT-2 cells was also performed. First, the control (Cr) or M-Sec knockdown (MSec-KD) MT-2 cells were analyzed for the expression of M-Sec protein by using western blotting (upper left). The results of densitometric analysis of the bands are shown. They were then serially passaged. In each passage, the cells were cultured for 3 days, and cell numbers were enumerated using the trypan blue dye exclusion method (lower panels). After 4th passage, the cells were re-analyzed for the expression M-Sec protein by western blotting (upper right). (PDF) [file ppat.1010126.s009.pdf]

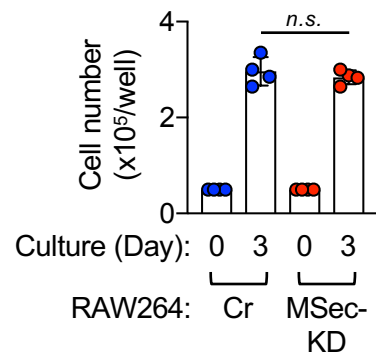

S9 Fig

Supplement: S9 Fig — The control (Cr)- or M-Sec knockdown (MSec-KD) RAW264 cells were provided by H. Ohno (RIKEN, Japan) [3]. The cells were seeded (0.5 x 10^5^ cells/well) and cultured with RPMI 1640/10% FCS for 3 days, and cell numbers were enumerated using the trypan blue dye exclusion method (n = 3). n.s., not significant. (PDF) [file ppat.1010126.s010.pdf]

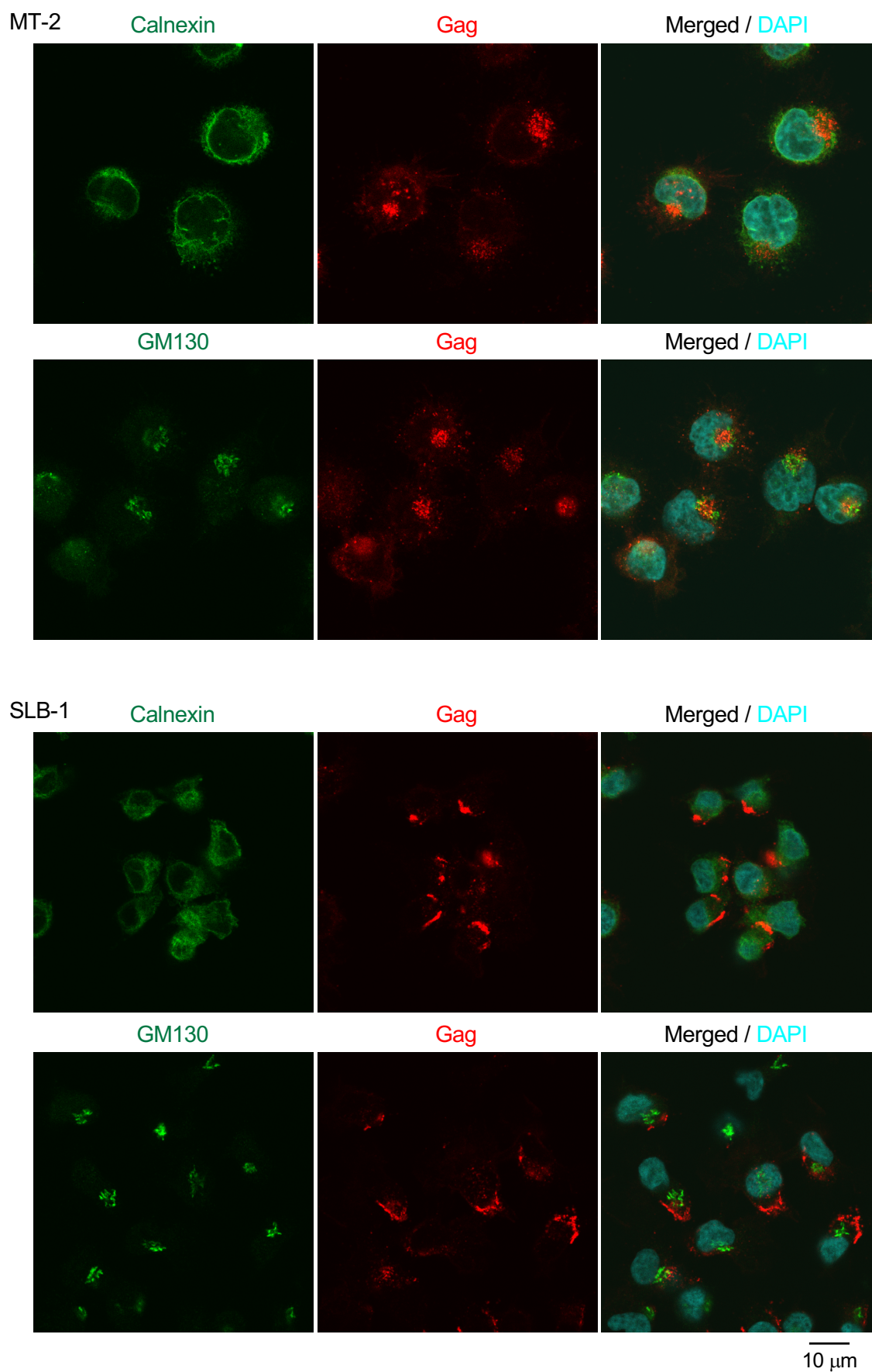

S10 Fig

Supplement: S10 Fig — The control MT-2 cells (upper panels) or SLB-1 cells (lower panels) were analyzed for Gag, Calnexin (as an endoplasmic reticulum marker), or GM130 (as a Golgi marker). The nuclei were also stained with DAPI. The antibodies used for staining were as follows: anti-Calnexin (C5C9; Cell Signaling Biotechnology), and GM130 (D6B1; Cell Signaling Biotechnology). Scale bar: 10 μm. (PDF) [file ppat.1010126.s011.pdf]

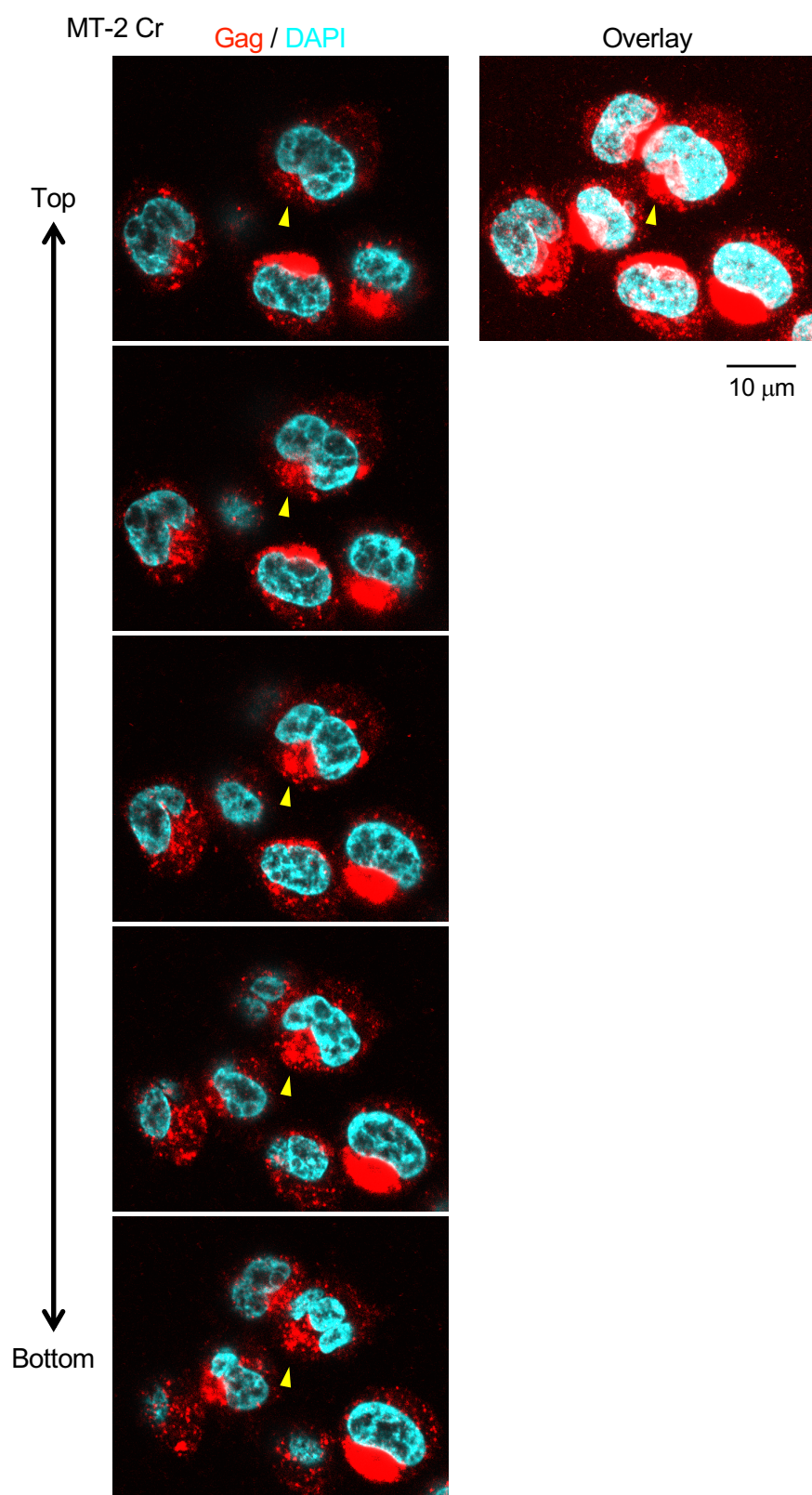

S11 Fig  
(related to Fig 8)

Supplement: S11 Fig — (related to Fig 8). The control MT-2 cells were stained as in Fig 8, and serial Z-sections from the top to the bottom are shown (left). An overlay image of the serial Z-sections is also shown (right). A yellow arrowhead indicates a typical large Gag cluster, which is composed of many puncta. Scale bar: 10 μm. (PDF) [file ppat.1010126.s012.pdf]

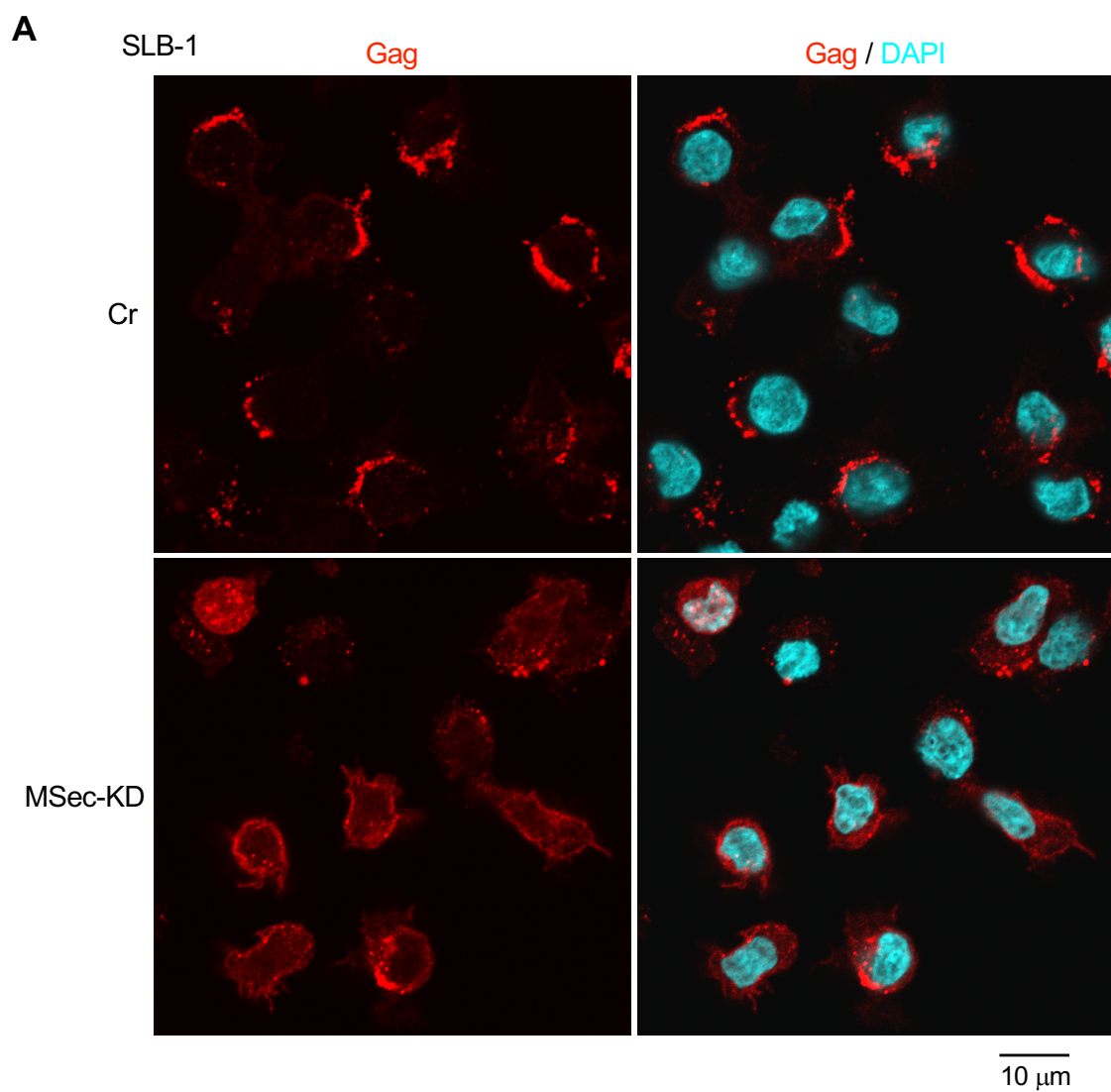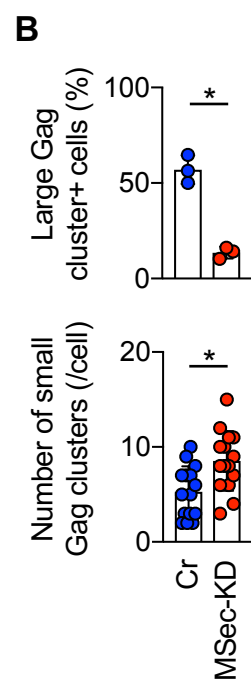

S12 Fig  
(related to Fig 8)

Supplement: S12 Fig — (related to Fig 8). (A) The control (Cr)- or M-Sec knockdown (MSec-KD) SLB-1 cells were analyzed for Gag (red). The nuclei were also stained with DAPI (blue). Scale bar: 10 μm. (B) The cells were analyzed as in (A). Three different fields were randomly selected, and the percentages of large Gag cluster+ cells were quantified (upper). The numbers of small clusters of Gag per cell are also shown (lower, 16 cells for each). *p < 0.05. (PDF) [file ppat.1010126.s013.pdf]

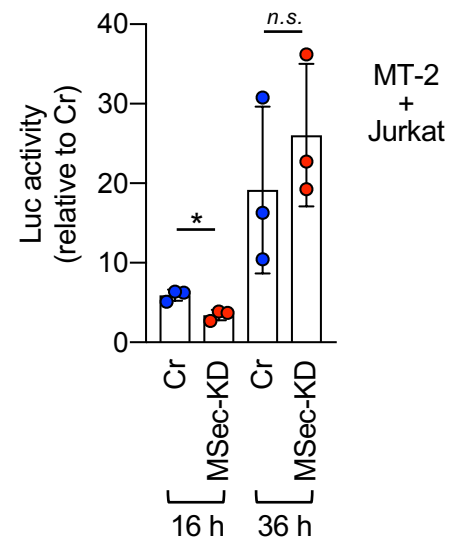

S13 Fig  
(related to Fig 4A)

Supplement: S13 Fig — (related to Fig 4A). Reporter Jurkat cells were co-cultured with control (Cr) or M-Sec knockdown (MSec-KD) MT-2 cells for 16 or 36 h. Luciferase activities are shown by setting the value of Jurkat alone as 1 (n = 3). *p < 0.05. n.s., not significant. (PDF) [file ppat.1010126.s014.pdf]

## Slide 1
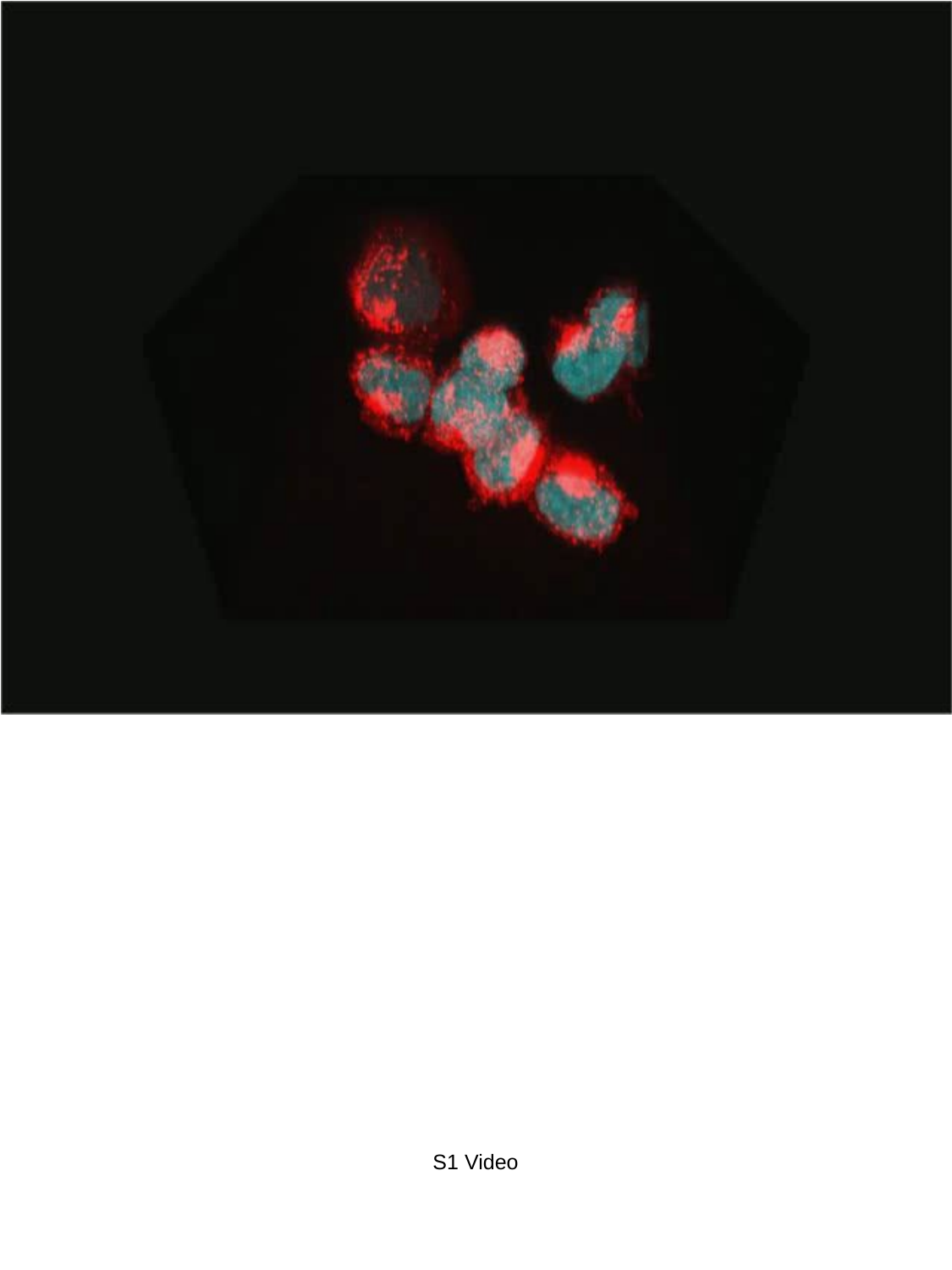

S1 Video

Supplement: S1 Video — The control MT-2 cells were analyzed for Gag (red). The nuclei were also stained with DAPI (blue). (PPTX) [file ppat.1010126.s015.pptx]

## Slide 1
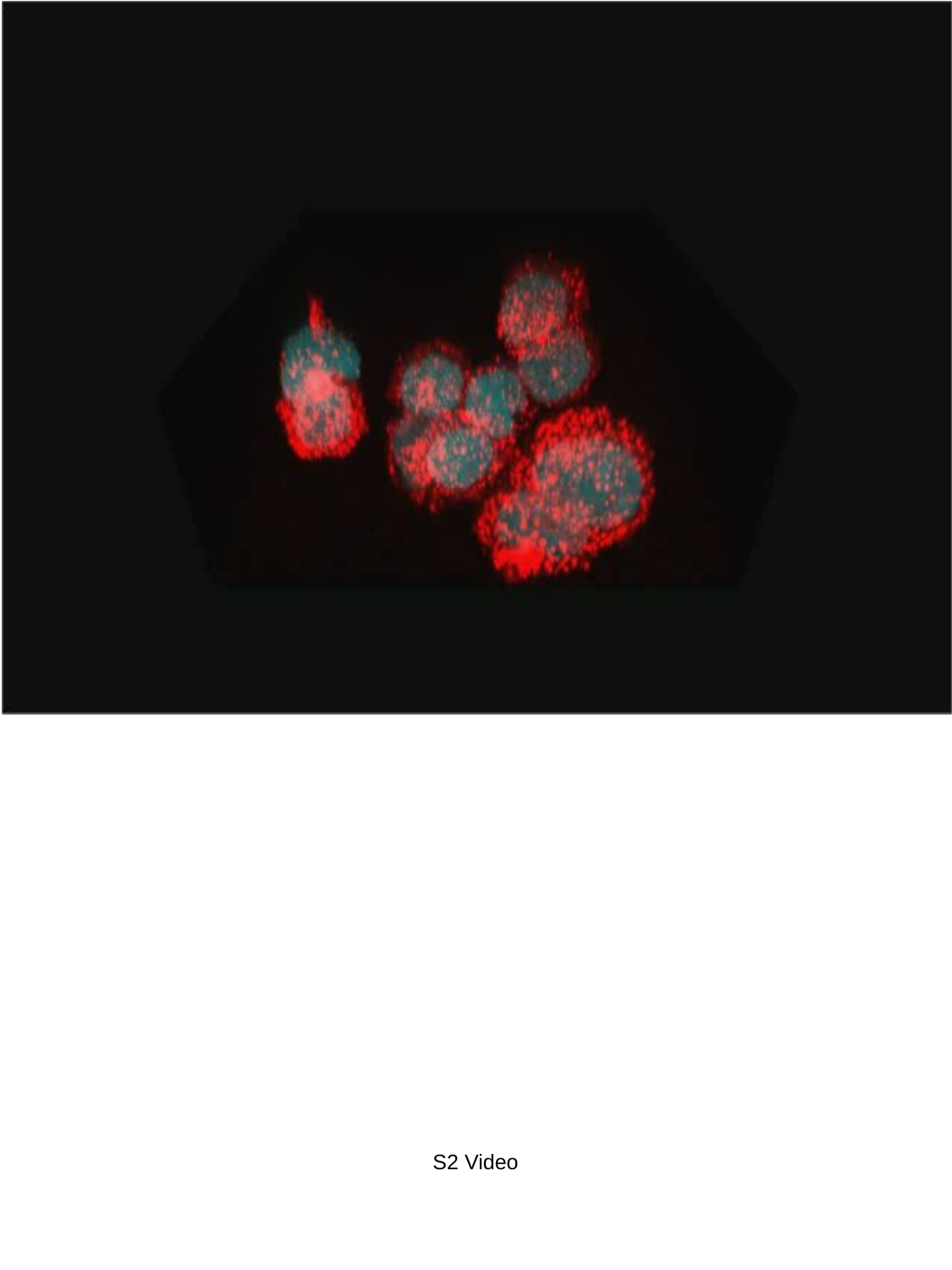

S2 Video

Supplement: S2 Video — M-Sec knockdown MT-2 cells were analyzed for Gag (red). The nuclei were also stained with DAPI (blue). (PPTX) [file ppat.1010126.s016.pptx]
